# The Virome of Cerebrospinal Fluid: Viruses Where We Once Thought There Were None

**DOI:** 10.3389/fmicb.2019.02061

**Published:** 2019-09-06

**Authors:** Chandrabali Ghose, Melissa Ly, Leila K. Schwanemann, Ji Hyun Shin, Katayoon Atab, Jeremy J. Barr, Mark Little, Robert T. Schooley, Jessica Chopyk, David T. Pride

**Affiliations:** ^1^Bioharmony Therapeutics, Inc., San Diego, CA, United States; ^2^Department of Pathology, University of California, San Diego, San Diego, CA, United States; ^3^School of Biological Sciences, Monash University, Melbourne, VIC, Australia; ^4^Department of Biology, San Diego State University, San Diego, CA, United States; ^5^Department of Medicine, University of California, San Diego, San Diego, CA, United States

**Keywords:** human microbiome, virome, virobiota, CSF, microbiota, body fluids, cerebral spinal fluid, cerebrospinal fluid

## Abstract

Traditionally, medicine has held that some human body sites are sterile and that the introduction of microbes to these sites results in infections. This paradigm shifted significantly with the discovery of the human microbiome and acceptance of these commensal microbes living across the body. However, the central nervous system (CNS) is still believed by many to be sterile in healthy people. Using culture-independent methods, we examined the virome of cerebrospinal fluid (CSF) from a cohort of mostly healthy human subjects. We identified a community of DNA viruses, most of which were identified as bacteriophages. Compared to other human specimen types, CSF viromes were not ecologically distinct. There was a high alpha diversity cluster that included feces, saliva, and urine, and a low alpha diversity cluster that included CSF, body fluids, plasma, and breast milk. The high diversity cluster included specimens known to have many bacteria, while other specimens traditionally assumed to be sterile formed the low diversity cluster. There was an abundance of viruses shared among CSF, breast milk, plasma, and body fluids, while each generally shared less with urine, feces, and saliva. These shared viruses ranged across different virus families, indicating that similarities between these viromes represent more than just a single shared virus family. By identifying a virome in the CSF of mostly healthy individuals, it is now less likely that any human body site is devoid of microbes, which further highlights the need to decipher the role that viral communities may play in human health.

## Introduction

For some time, many body surfaces were believed to be inhabited by few if any microorganisms and the exposure to bacteria, viruses, and other pathogens generally resulted in potentially life-threatening infections. The development of modern cultivation techniques (Emerson and Wilson, 2009; Stewart, 2012) and next-generation sequencing technologies (Goodwin et al., 2016; Cao et al., 2017) has revealed that these body surfaces are inhabited by a much broader array of microorganisms. These microbes collectively are referred to as the human microbiome. They are involved in important metabolic and physiological processes (Turnbaugh et al., 2009; Qin et al., 2010; Burke et al., 2011; Human Microbiome Project Consortium, 2012; Ursell et al., 2012), and are increasingly recognized for their role in human health (Fujimura et al., 2010; Willing et al., 2010; Relman, 2012; Arrieta et al., 2015). There is increasing recognition of microbes associated with the mouth, gut, skin, vagina, bladder, and lungs (Costello et al., 2009; Human Microbiome Project Consortium, 2012; Cui et al., 2014), but in other regions of the body such as the blood, and central nervous system (CNS), the presence of a microbiome is not widely recognized or accepted.

Much of the human microbiome research is focused on the bacterial component, yet a growing body of work is demonstrating the ubiquity of viral communities across the body. Studies have shown the presence of viral communities in saliva and dental plaque (Abeles et al., 2014; Ly et al., 2014), lower gastrointestinal (GI) tract (Reyes et al., 2010; Columpsi et al., 2016; Thannesberger et al., 2017), respiratory tract (Wylie, 2017), skin (Hannigan et al., 2015), bladder (Santiago-Rodriguez et al., 2015a), vagina (Wylie et al., 2014, 2018), bloodstream (Sauvage et al., 2016; Moustafa et al., 2017), and breast milk (Pannaraj et al., 2018). The abundance of viruses found across the body suggests that there may be no body surfaces that are truly devoid of viruses; thus, no body surface may be truly sterile.

The majority of the viruses found on human body surfaces may be bacterial viruses known as bacteriophages. On some of these surfaces, bacteriophages (phages for short) are persistent and ubiquitous, with some able to persist months and perhaps years (Minot et al., 2011; Abeles et al., 2014; Ly et al., 2016). Members of phage communities can be shared with unrelated housemates (Robles-Sikisaka et al., 2013; Ly et al., 2016), suggesting that they may not just affect the bacteria on your body surfaces, but that they may affect the bacterial communities of people you live with as well. The ecology of phage communities appears to be specific for certain body sites. For example, the phage communities of GI tract are unique from those found in the mouth (Abeles et al., 2015). Further site-specific features have been observed in the oral cavity with salivary phages differing significantly from those found within dental plaque (Ly et al., 2014). In certain disease conditions, including periodontal disease (Ly et al., 2014) and ulcerative colitis (Norman et al., 2015), phage communities are associated with the disease condition. Yet whether these phages play a role in disease development or maintenance is not yet known.

In fact, we know very little about the role and function of viromes across the human body. Due to their abundance, phages have been hypothesized to shape bacterial communities by killing their hosts (Edlund et al., 2015). Bacteriophages may provide non-host-derived immunity by binding to human mucosal layers, regulating mucosal bacterial communities via subdiffusive motion, which increases their probability to contact with a bacterial host (Barr et al., 2013, 2015). Recently, phages were demonstrated to transcytose across *in vitro* epithelial barriers (Nguyen et al., 2017). Bacteriophage transcytosis from the gut may provide a mechanism for phages to directly access the internalized body sites not previously known to harbor viral communities.

In this study, we sought to evaluate whether the traditionally “sterile” CNS in humans contains a stable virome. We obtained cerebrospinal fluid (CSF) from a cohort of 20 subjects with and without infections, and contrasted these with viromes obtained from a number of different body sites. Our goals were to: (1) demonstrate the presence of a virome unique to the CNS, (2) identify whether viruses found in CSF are similar to those found on other body surfaces, and (3) identify ecological trends in CSF viromes and compare with ecological trends observed on other body surfaces.

## Materials and Methods

### Human Subjects and Culture Conditions

Human subject involvement in this study was approved by the University of California, San Diego Administrative Panel on Human Subjects in Medical Research. The study was certified as category 4 exempt, which does not require informed consent on behalf of the study subjects. The research was categorized as exempt because it involved the collection or study of existing data, documents, records, pathological specimens, or diagnostic specimens that were recorded in such a manner that the subjects could not be identified, directly or through identifiers linked to the subjects. We sampled CSF from 20 human subjects, body fluids from 5 subjects, and plasma (the supernatant collected after spinning whole blood centrifuged for 15 min that was collected in EDTA-treated lavender top tubes) from 10 subjects (Supplementary Table S1). Each of the CSF, plasma, and body fluid specimens was stored at 4°C, and processed within 7 days of their collection.

CSF specimens were collected under sterile conditions using standard protocols (Teunissen et al., 2009). Briefly, skin was sterilized with povidone-iodine and allowed to dry. The lumbar area was draped to create a sterile field, and a 22 gauge needle was inserted between the L3 and L5 vertebral bodies until CSF began to flow. The first few drops were discarded, and 4 separate tubes of CSF were collected. Approximately 2–4 mL of CSF was collected into each tube consecutively from Tube#1 to Tube#4. The CSF that was collected in Tube#3 was used for standard of care cultures, and then was used for virome analysis in this study. Tubes#1 and #4 were used for cell counts, and counts from Tube#4 are reported in this study (Supplementary Table S1). Standard of care procedures for CSF collection were followed for all subjects enrolled in the study. Because this study was certified as category 4 exempt, the study team could not be involved in the CSF collection process.

### Assessing the CSF and Body Fluids for Bacteria

In an effort to verify that CSF specimens were relatively *devoid* of bacteria, we analyzed the CSF specimens for the presence of 16S rRNA. DNA was extracted from CSF using the Qiagen DNeasy Powersoil kit (Qiagen) and further concentrated using the Zymo gDNA Clean and Concentrate kit (Zymo). Purified and concentrated DNA was subjected to PCR using Kapa Hifi Hotstart Readymix (Kapa Biosystems) and PCR forward primer 5′-TCG TCG GCA GCG TCA GAT GTG TAT AAG AGA CAG CCT ACG GGN GGC WGC AG-3′ and reverse primer 5′-GTC TCG TGG GCT CGG AGA TGT GTA TAA GAG ACA GGA CTA CHV GGG TAT CTA ATC C-3′ to amplify the V3–V4 hypervariable segment of 16S rRNA. We used the following cycling parameters: 95°C for 3 min, followed by 35 cycles of 95°C for 30 s, 55°C for 30 s, 72°C for 30 s, and a final elongation step of 72°C for 5 min. Amplicons were purified with Ampure XP beads (Beckman-Coulter) and visualized using a High Sensitivity DNA Kit on a Bioanalyzer (Agilent Technologies). No amplification products could be visualized from CSF specimens from subjects not already known to be infected with bacteria.

### Virome Preparation and Sequencing

CSF and body fluid specimens were treated in an identical manner as we have developed for processing of saliva specimens (Abeles et al., 2014). To determine the counts of virus-like particles in CSF and body fluids, we modified an existing procedure commonly used to isolate viruses from environmental samples (Thurber et al., 2009). Samples were filtered sequentially using 0.45 and 0.2 μm cellulose acetate filters (GE Healthcare Life Sciences) to remove cellular and other debris. A 10 μL aliquot of the filtered CSF or body fluid was resuspended in 190 μL of 0.02 μm filtered PBS, and was then filtered through a 0.02 μm filter to trap the virus particles. They were then stained using SYBR-gold and visualized by epifluorescence microscopy (Noble and Fuhrman, 1998). The concentration of the virus-like particles was estimated based on the mean number of particles from at least four separate high-power fields.

To purify the CSF and body fluid viromes, the samples were filtered sequentially using 0.45 and 0.2 μm cellulose acetate filters (GE Healthcare Life Sciences) to remove cellular and other debris (Pride et al., 2012). CSF and body fluid specimens were then purified on a cesium chloride gradient according to our previously described protocols (Pride et al., 2012). Only the fraction with a density corresponding to most known bacteriophages (Murphy et al., 1995) was retained, further purified on Amicon YM-100 protein purification columns (Millipore, Inc.), treated with 2 units of DNase I, and subjected to lysis and DNA purification using the Qiagen UltraSens Virus kit (Qiagen). Recovered DNA was screened for the presence of contaminating bacterial nucleic acids by the protocol described above. Viral DNA then was amplified using GenomiPhi Hy MDA amplification (GE Healthcare Life Sciences), and specimens prepared for sequencing using the Nextera DNA Library Prep XT kit (Illumina, Inc.) according to manufacturer instructions. We included sterile water that had gone through the virome extraction process as negative controls for the MDA amplification, and were able to produce amplified products from the sterile water. Those specimens were further processed identically to the other virome specimens. The size of amplified virome products was determined using a High Sensitivity DNA Kit on a Bioanalyzer (Agilent Technologies), and quantified using a High Sensitivity Double Stranded DNA kit on a Qubit Fluorometer (Thermo Fisher Scientific). DNA from each specimen was pooled into equimolar proportions and sequenced on the Illumina MiniSeq Instrument (Illumina, Inc.). We trimmed sequence reads according to Phred scores of 30, removed any low complexity reads with ≥8 consecutive homopolymers, and removed any reads with substantial length variation (<50 nucleotides or >300 nucleotides) or ambiguous characters prior to further analysis. Each virome was screened for contaminating nucleic acids using BLASTN analysis (E-score < 10^–5^) against the human reference database available at ftp://ftp.ncbi.nlm.nih.gov/genomes/H_sapiens/. Any reads with significant sequence similarities to human sequences were removed prior to further analysis using Ion Assist^1^. We also screened the virome reads for potential bacterial contamination by mapping reads with low stringency (80% similarity across 20% of the read length) against the database of bacterial genomes^2^ using CLC Genomics Workbench 9.5.3 (Qiagen). For any virome where >10% of the reads mapped to bacteria genomes, we manually examined the read mapping patterns. When viromes mapped across much of the bacteria genome, they were considered contaminated and were removed from the study even if bacteria were not detected by 16S rRNA amplification.

### Virome Analysis

All reads were assembled using CLC Genomics Workbench 9.5.3 (Qiagen) based on 98% identity with a minimum of 50% read overlap, which were more stringent than those developed to discriminate between highly related viruses (Breitbart et al., 2002). Because the average and median read lengths were 150 nucleotides, the minimum tolerable overlap was approximately 75 nucleotides (Supplementary Table S2). We also constructed contigs based on 98% identify with a minimum of 80% read overlap. Because the constructed contigs were highly similar to those constructed with a minimum of 50% read overlap (Supplementary Table S3), we used those constructed at 50% read overlap to be consistent with our prior studies. The consensus sequence for each contig was constructed according to majority rule and any contigs <200 nucleotides were removed prior to further analysis.

Virome contigs were annotated using BLASTX against the NCBI NR database with an *E*-value cutoff value of 10^–5^. Specific viral sequences were identified using Ion Assist^3^ by parsing BLASTX results for known viral genes including replication, structural, transposition, restriction/modification, hypothetical, and other genes previously found in viruses for which the *E-*value was at least 10^–5^. Each individual virome contig was annotated using this technique, however, if the best hit for any portion of the contig was to a gene with no known function, lower level hits were used as long as they had known function and still met the *E*-value cutoff. These BLASTX hits were used for comparisons of gene functions in viruses, but not for taxonomic analysis. ORF prediction was performed using FGenesV (Softberry Inc., Mount Kisco, NY, United States), and putative functions assigned by BLASTP homology against the NR database (Escore < 10^–5^). Virus types were determined by parsing the virus families from the TBLASTX best hits of each viral contig with an *E*-value < 10^–20^ (Pannaraj et al., 2018). We also identified virome contigs with significant homologs in the IMG/VR v2.0 database (Paez-Espino et al., 2019) using BLASTX with an *E*-value cutoff value of 10^–5^.

We constructed assemblies from the CSF contigs of all subjects combined using 98% identity across 50% of the read lengths to determine viruses that might be present in multiple different subjects. We then characterized the resulting assemblies to determine which subjects contributed to the construction of these larger viruses and the relative coverage of each contig from each subject. We characterized these larger viral assemblies using BLASTX analysis (*E*-value cutoff 10^–5^) against the NR database. Resulting virus structures were determined for ORF structures and directionality using MetaGeneMark (Zhu et al., 2010). ORFs were annotated on each contig with Geneious v. 11.1.5 (Kearse et al., 2012).

Analysis of shared sequence similarities present in each virome was performed by creating custom BLAST databases for each virome, comparing each database with all other viromes using BLASTN analysis (*E*-value < 10^–10^), and these compiled data used to calculate beta diversity with Bray Curtis distances using QIIME (Caporaso et al., 2010). These distances were used as input for PCoA. Statistical tests were performed using the Adonis function in the R Vegan package with 999 permutations (Oksanen et al., 2010). We determined putative sharing of homologous viruses by constructing assemblies from all specimen types, and then deciphered the contribution to each resulting contig, similar to techniques we have previously described (Abeles et al., 2014; Ly et al., 2016). We utilized this technique to decipher those contigs that were unique to each specimen type, and those shared between different specimen types. Statistical significance was determined by comparisons between groups by the Mann–Whitney *U*-test using SPSS 25 (IBM). We also performed a permutation test (10,000 iterations) to assess whether viromes from different specimen types had significant overlap using Ion Assist^4^ (Robles-Sikisaka et al., 2013). Included among these specimens that we examined from prior studies was saliva from 8 subjects, feces from 9 subjects, urine from 20 subjects, and breast milk from 10 subjects (Robles-Sikisaka et al., 2013; Abeles et al., 2014; Ly et al., 2014; Naidu et al., 2014; Robles-Sikisaka et al., 2014). We simulated the distribution of the fraction of shared virome homologs between all specimen types. For each specimen type, we computed the summed fraction of shared homologs using 1000 random contigs between randomly chosen between specimen types, and from these computed an empirical null distribution of our statistic of interest (the fraction of shared homologs). The simulated statistics within each specimen type was referred to the null distribution of inter-specimen type comparisons, and the *p*-value was computed as the fraction of times the simulated statistic for the each exceeded the observed statistic.

### Viral Diversity

To measure alpha diversity in the viral communities, we utilized a technique termed the Homologous Virus Diversity Index (HVDI). The technique is based on finding high levels of homology amongst contigs within viromes that likely belong to the same virus but were placed into separate contigs due to the limitations of the assembly process (Abeles et al., 2014). Virome reads were assembled using 98% identify over a minimum of 50% of the read length using CLC Genomics Workbench 9.5.3 (Qiagen), and the resulting contig spectra utilized as the primary input for the index. We did not use any contig length cutoff for our analysis of alpha diversity because the metrics for determining alpha diversity are highly dependent on the number of singletons (reads that did not assemble) in their estimates of diversity. We created custom nucleotide BLAST databases for each specimen type that contained all contigs. We then used BLASTN analysis to find high levels of homology (e-score < 10^–20^) between different contigs within the same specimen type. We accepted only high levels of homology that spanned at least 50% of the length of the shorter contig being compared, which would be a minimum length of 75 bp. All contigs in each specimen type were treated as nodes and those contigs that had high homology to other contigs in the same specimen type were added to a network by directing edges between the nodes. After evaluating homologies among all intra-specimen type contigs, networks formed from directed edges/nodes were assigned to individual viruses and nodes with no associations were considered singular viruses. For each resulting network, we added the number of reads assigned to each node on the network and the combined number of reads was used to represent the relative abundance of the virus represented by that network. The relative abundances of all viruses were calculated using this technique, and a new contig spectrum representing the viral population in each specimen type was formed. The contig spectrum from each specimen type then was used as a surrogate for population structures and input directly into the chao1 index (Chao, 1984) to estimate diversity.

## Results

### Human Subject Characteristics

We received approval from our Human Subjects Protections Program to obtain residual CSF specimens from a cohort of 20 subjects who had undergone lumbar punctures within the prior 72 h. Of those 20 individuals, four subjects were diagnosed with CNS infections based on their CSF white blood cell counts and the recovery of pathogens (Supplementary Table S1). We also obtained residual specimens from other specimen types, including blood plasma from 10 human subjects, and miscellaneous body fluids from another five subjects (Supplementary Table S1). These specimens were also collected within the prior 72 h. Of those five miscellaneous body fluid samples, three specimens were peritoneal fluid, one was hip fluid, and one sample was bile. The biliary fluid specimen was collected from a drainage tube in an individual with cholangitis (an infection of the bile duct), while the other body fluid specimens were clinically sterile (no growth on standard culture media) (Supplementary Table S1).

### Processing of CSF and Body Fluid Viromes

We performed epifluorescence microscopy on CSF specimens to identify the presence of virus-like particles (VLPs) prior to virome processing. We found an average of 10^4^ VLPs per mL of CSF in the cohort (Supplementary Figure S1A), compared with non-sterile saliva samples from another cohort, which contained to 10^6^ VLPs (Supplementary Figure S1B). These data indicate that there were VLPs present within the CSF, but at many orders of magnitude less than a traditionally non-sterile site. We next processed the DNA viromes isolated from CSF, plasma, and other body fluids according to our previously described protocol for virome preparations from saliva (Abeles et al., 2014). Briefly, samples were sequentially filtered, purified via cesium chloride density gradient centrifugation, followed by lysis of virions, extraction of nucleic acids, and next-generation sequencing. We also processed viromes from sterile water to help determine whether viromes could be contaminated from viruses that are observed in the water used in the experiments. From the 20 CSF specimens, we sequenced 41,102,484 reads of mean length 150 nucleotides for an average of 2,055,124 reads per subject; for the 10 plasma specimens, we sequenced 10,694,439 reads of mean length 150 nucleotides for an average of 1,069,439 reads per subject; for the 5 body fluid specimens, we sequenced 5,594,438 reads of mean length 150 nucleotides for an average of 1,118,888 reads per subject (Supplementary Table S2). We extracted DNA from a subset of 10 CSF specimens without clinical infections to amplify and sequence the V3–4 segment of 16S rRNA, but no amplicons could be obtained when measured on a bioanalyzer. Our inability to amplify 16S rRNA from CSF specimens suggests that they were relatively *devoid* of bacteria.

We assembled virome reads from each subject and specimen type to build larger viral contigs because their greater length leads to more productive searches for homologous sequences in available databases (Pride et al., 2012). We obtained a mean of 273 ± 25 contigs per CSF specimen, 143 ± 33 contigs per body fluid specimen, 220 ± 25 contigs per plasma specimen (Supplementary Table S2), and 162 ± 25 for water controls (Supplementary Figure S2). Most of the CSF reads assembled into contigs that had homology to viruses (70.6 ± 4.6%), bacteria (15.6 ± 3.1%); seen commonly when lysogenic phages are found in viromes (Pride et al., 2012), or had no known homologies (13.8 ± 3.6%; Supplementary Figure S3). Plasma reads assembled into contigs with 78.8 ± 5.9% homologous to known viruses, 18.6 ± 5.9% homologous to bacteria, and 11.2 ± 3.5% with no known homologs. Body fluid reads assembled into contigs with 60.8 ± 4.9% homologous to known viruses, 12.0 ± 3.4% homologous to bacteria, and 27.3 ± 6.0% with no known homologs. From the sterile water, we also were able to identify 21% with homology to known viruses, none with homology to bacteria, and 79.2% with no known homologies. Of those 20.8% with homologs to viruses, 19.2% were homologous to an *E. coli* Lambda phage that was not also identified in CSF specimens. These findings combined reveal substantial virus populations in CSF, body fluids, and plasma.

We also examined the CSF viromes using the IMG/VR v2.0 database (Paez-Espino et al., 2019). The IMG/VR v2.0 database is currently the largest available dataset available for virome analysis, and includes cultivated viruses, curated prophages, and computationally predicted viruses from metagenomes. We found that 68.3 ± 0.7% of the CSF viromes had homologs in this database (Supplementary Figure S4) with median E-score values ranging from 3^–27^ to 6^–38^, which was similar to what we observed with the NR database (Supplementary Figure S3). Many of the viral contigs (70.3 ± 1.1%) from body fluids were also homologous to virus metagenome fragments (Supplementary Figure S4). We also characterized the habitats from which these homologs were derived (Supplementary Figure S5). A large proportion of them were homologous to curated prophages (16.5%), human associated viromes (15.7%), aquatic viromes (24.4%), plant viromes (13.4%), and viromes from the built environment (9.51%) (Supplementary Table S4). Of those human associated viromes, 7.8% were homologous to fecal viromes and 6.7% were homologous to oral viromes. Only a small percentage (0.8%) had homologs identified in viromes from the skin.

### Beta Diversity in CSF and Body Fluid Viromes

We characterized the beta diversity in the viromes of CSF and body fluids using Bray Curtis distances, tested the significance with Adonis, and visualized the output using PCoA analysis. While significant (*p* < 0.05, *R*^2^ = 0.07) only a small percentage of the variation could be explained by the sample type (Supplementary Figure S6). We next analyzed the beta diversity amongst the CSF specimens to identify whether there may be distinguishing features between those individuals with infections compared to those without. While we only characterized specimens from 4 individuals with CNS infections (Supplementary Table S1), there were no trends in beta diversity that distinguished these specimens (data not shown). These findings suggest that infection status may not be a characteristic that defines CSF viral ecology, but a larger sample size is necessary to confirm this finding.

### Taxonomic Analysis of Viruses in CSF, Body Fluids, and Other Human Specimen Types

We next characterized the putative taxonomic compositions of the CSF and body fluid viromes to determine whether these viromes were populated by different families of viruses. Most of the contigs (93.4 ± 0.3% for CSF and 89.4 ± 2.3% for body fluids) could not be assigned to viral families (Supplementary Figure S7). Of those contigs that could be assigned, the vast majority were bacteriophages in body fluids (96.3 ± 2.5%) and CSF (83.1 ± 5.1%), while fewer eukaryotic virus families were identified in each sample type. The majority of the bacteriophages identified were Caudovirus families *Myoviridae* (generally larger dsDNA genomes with contractile tails), *Siphoviridae* (mid-sized dsDNA genomes with non-contractile tails), and *Podoviridae* (smaller dsDNA genomes with short tail stubs) (Supplementary Figure S8). Many but not all siphoviruses have primarily lysogenic lifestyles, while myoviruses and podoviruses often have lytic lifestyles (Wichels et al., 1998; Sullivan et al., 2003). Phages from the ssDNA family Inoviridae and other dsDNA phages that could not be further classified were also identified. Amongst the eukaryotic viruses identified were *Phycodnaviridae*, *Herpesviridae*, and *Mimiviridae*. We failed to identify differences in the CSF that might delineate the infected from the uninfected individuals. These data suggest that many different virus families inhabit CSF and body fluids, with bacteriophages representing the preponderance of the community.

**FIGURE 1:**
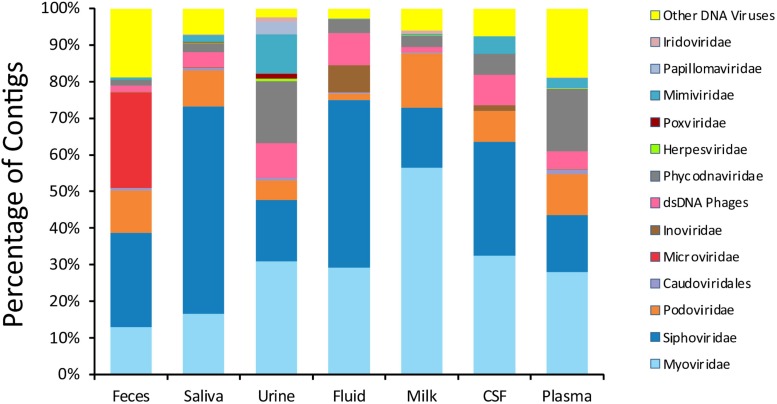
Proportion of viral contigs with TBLASTX hits to the specified virus families. The y-axis represents the percentage of contigs homologous to each family, or that were unclassified dsDNA phages or other viruses. The sample type is shown on the x-axis. The percentage of reads was determined based on the raw number of reads used to assemble each contig.

To delineate whether taxonomic trends seen in CSF and body fluid viromes may be similar to those from other human body sites, we compared data from previously sequenced viromes from feces (Abeles et al., 2015), saliva (Abeles et al., 2014), urine (Santiago-Rodriguez et al., 2015a), and breast milk (Pannaraj et al., 2018) in our prior studies. There were some features that delineated many specimen types (Figure 1). In feces, there was an abundance of microviruses; in saliva there were high levels of siphoviruses; in urine, there were papillomaviruses and herpesviruses; in breast milk, there were substantial numbers of myoviruses; in body fluids, there were many viruses from the phage family *Inoviridae*. There were no obvious taxonomic distinguishing features of CSF or plasma viromes that could not be identified in the other specimen types.

### Diversity Amongst CSF and Other Specimen Types

**FIGURE 2:**
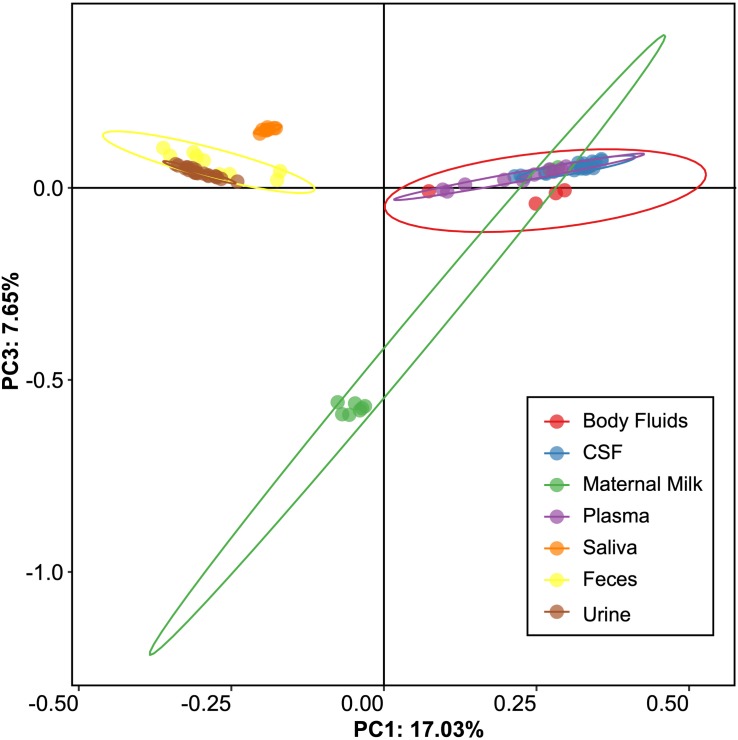
Representation of beta-diversity based on Bray Curtis distances, shown in Principal coordinates analysis of the viral communities. Ellipses are drawn at 95% confidence intervals for sample type.

We next examined beta and alpha diversity in the viromes of each specimen type. We visualized beta diversity using PCoA analysis and tested whether there were significant differences among the sample types with Adonis (Figure 2). The viral communities were distinct based on sample type (*p* < 0.001, *R*^2^ = 0.37). However, most of the variation was observed along coordinate 1, where the viromes of urine, saliva, and feces shared similarities. CSF, plasma, and body fluid specimens also clustered similarly on coordinate 1. The breast milk specimens were located between these separate clusters, and clustered separately from all other specimen types along coordinate 3.

We also performed a permutation test to decipher whether the differences observed by PCoA could be further supported statistically (Table 1). We found that saliva and feces each could be differentiated from other specimen types, but that there were no statistically significant patterns amongst the other specimen types. These data indicate that there are significant differences amongst some specimen types, but most have shared features that render them difficult to distinguish.

**TABLE 1:** Viral homologs within and between specimen types.

**By subject**	**Percent homologous within sample type^a^**	**Percent homologous between sample types^a^**	***P*-value^b^**
CSF	17.38 ± 0.02	4.89 ± 0.07	0.0984
Body fluids	6.78 ± 0.03	5.32 ± 0.06	0.3878
Milk	17.00 ± 0.15	2.83 ± 0.06	0.1870
Plasma	11.20 ± 0.08	6.97 ± 0.09	0.3033
Stool	19.18 ± 0.12	2.00 ± 0.03	**0.0382**
Saliva	54.42 ± 0.05	1.76 ± 0.04	**<0.0001**
Urine	9.89 ± 0.08	1.77 ± 0.04	0.0925

*^*a*^Based on the mean ± standard deviation of 10,000 iterations. 10,000 random contigs were sampled per iteration; ^*b*^P-value based on the fraction of times the estimated percent homologous contigs within each sample type exceeds that for different sample types. *p*-values <= 0.05 are represented in bold.*

**FIGURE 3:**
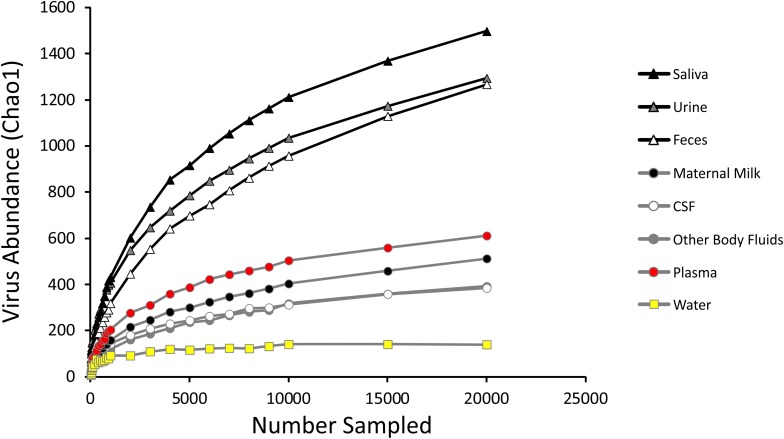
Homologous Virus Diversity Index rarefactions based on the Chao1 Index of viral communities from various specimen types. The x-axis represents the number of virome reads sampled, and the y-axis represents virus abundance.

We measured alpha diversity in the viromes to discern whether there were differences in the relative numbers of virus genotypes and their distributions. We characterized alpha diversity using the Homologous Virus Diversity Index (HVDI) (Santiago-Rodriguez et al., 2015b) based on the chao1 index (Chao, 1984), which takes into account the abundance of viruses and their distribution, but also accounts for lesser abundant viruses that may have been missed due to under sampling. There was a significant (*p* < 0.001) contrast in the alpha diversity observed in the viromes of urine, saliva, and feces when compared to breast milk, body fluids, plasma, and CSF. Urine, saliva, and feces were of relatively high diversity, while breast milk, body fluids, plasma, and CSF were of relatively low diversity (Figure 3). The alpha diversity found in viromes from the sterile water controls was substantially lower than all the other viromes from the various different body sites. We also examined the alpha diversity using the Shannon index and found similar results, with the exception that the fecal viromes had much lower diversity (Supplementary Figure S9). These data indicate that there are significant differences in the viral ecology of some body sites, with breast milk, CSF, plasma and body fluids belonging to a low diversity ecological cluster, and saliva, feces, and urine belonging to a high diversity cluster.

### Highly Homologous Viruses in CSF and Other Specimen Types

**FIGURE 4:**
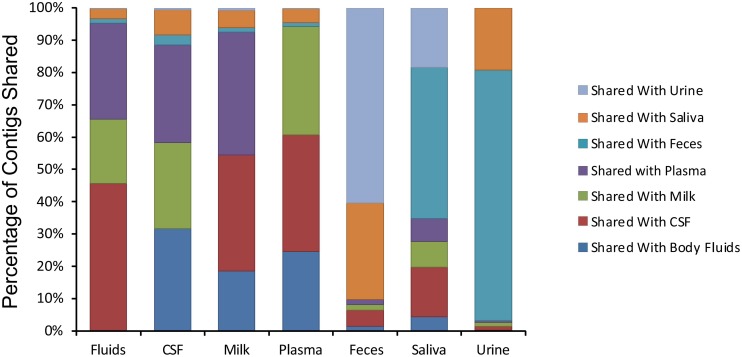
Percentage of contigs that were used in the assembly of larger viruses from various human specimens. The y-axis represents the percentage of viruses assembled that contained contigs from various specimen types, and the x-axis represents each specimen type. For example approximately 45% of viruses assembled from body fluid viromes also contained contigs from cerebrospinal fluid.

We next examined whether there may be similar homologous viruses within the ecological clusters. To examine these similarities, we assembled contigs from all specimen types and then measured the proportion of the resulting assemblies that were derived from each specimen type. We found that the majority of the contigs assembled from body fluids also included contributions from CSF, breast milk, and plasma (Figure 4). Few of the contigs assembled from body fluids included any contributions from urine, saliva, or feces. A similar trend was seen for CSF viromes that primarily assembled with body fluid, plasma, and breast milk viromes; breast milk and plasma viromes also followed this trend. A different trend was observed in feces, saliva, and urine, where they primarily assembled with each other. While the majority of the assemblies including saliva also included feces and urine, a number of saliva assemblies also included contributions from CSF, milk, and plasma. These results help to solidify that the body surfaces can be grouped into two separate clusters, one containing urine, feces, and saliva, and another that includes CSF, plasma, body fluids, and breast milk.

**FIGURE 5:**
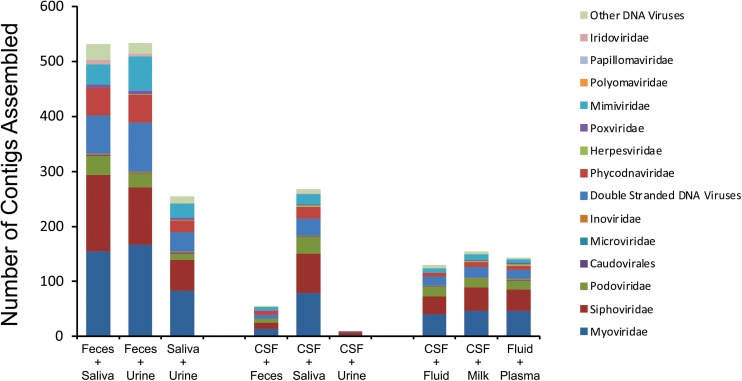
Putative virus family assignments of contigs assembled from various human specimen types. The y-axis represents the raw number of contigs assembled assigned to each virus family, and the x-axis represents contigs assembled from specific viromes.

We next characterized the homologous viruses shared between specimen types to determine whether they were shared from a single family or across multiple different families. We found that there was no single virus family that was shared within or between the different ecological clusters; instead, we found all observed virus families shared across the specimen types (Figure 5). For example, myoviruses, siphoviruses, and podoviruses, etc. were all shared among feces, urine, and saliva, but each of these virus types were also shared among breast milk, CSF, and body fluids. Very little sharing was observed between the two separate ecological clusters (Figure 5), with the exception of the aforementioned sharing between CSF and saliva (Figure 4). Our results including the sharing of homologous viruses, the grouping of viromes according to their high or low relative alpha diversity, and the clustering observed on the PCoA analysis, confirm the presence of ecological clusters among viromes of different body sites and demonstrate that the clusters are formed through the sharing of viruses from multiple different families.

### Individual Viruses

We characterized the structures of some of the shared homologous viruses among the different body sites to identify characteristics of some virus genomes that are shared within ecological clusters. Viral genomes were constructed by assembling contigs with high stringency among all of the different body sites. We identified Contig1516 that was assembled exclusively with contributions from urine, feces, and saliva (Supplementary Figure S10). Interestingly, we did not identify this virus in our prior study (Abeles et al., 2014), however, we could identify the entire 56 kb viral genome from 7 of the 8 subjects in that study. While we could identify the entire structure from saliva, we could only identify portions in 7 of the 9 fecal and 4 of the 20 urine viromes. This virus has significant homology to other podoviruses similar to phi29. We also identified Contig20 that was identified in 4/6 body fluid specimens and in 20/20 CSF specimens (Supplementary Figure S11). This virus has sequence similarities to other myoviruses, but has integrase and repressor homologs, indicating that it likely has a primarily lysogenic lifestyle. These data help to verify the existence of shared membership among the viromes within the distinct ecological clusters.

**FIGURE 6:**
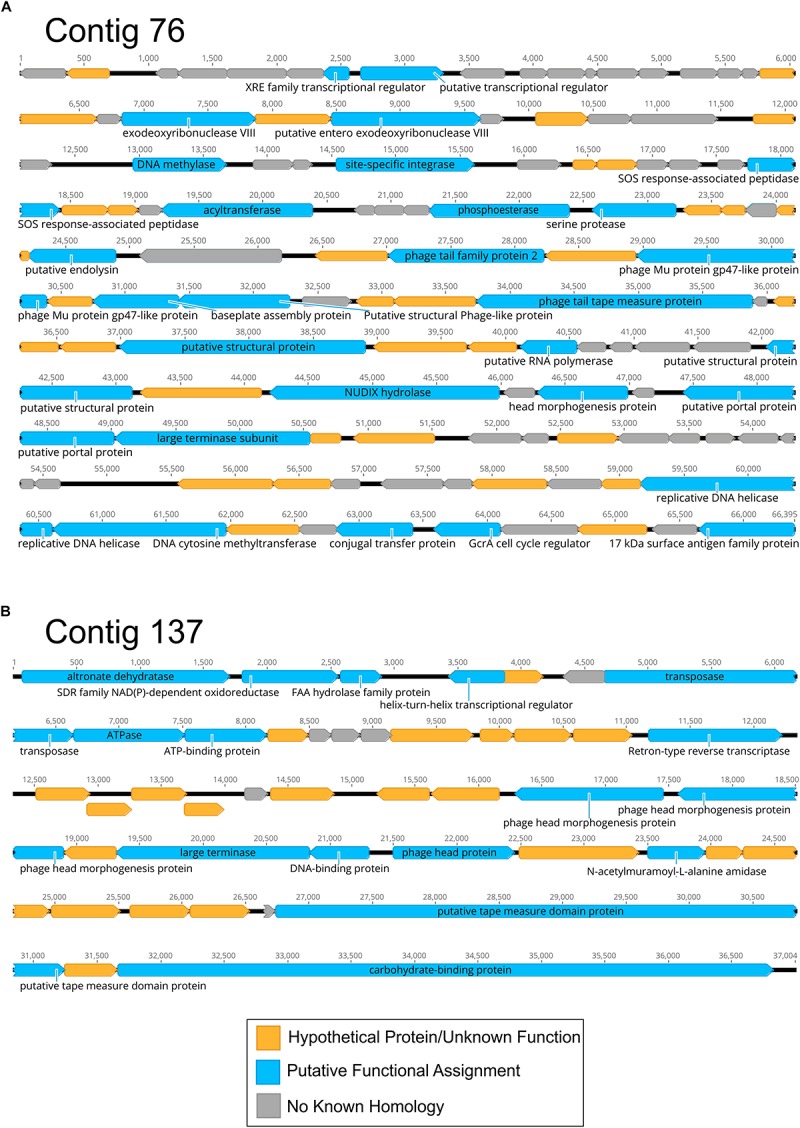
Diagram of Contigs 76 **(A)** and 137 **(B)** assembled from CSF viromes of all 20 subjects. Putative ORFs and their direction are indicated by the arrow boxes. ORFs that had significant homologs (BLASTX *E* < 10^–5^) are shown in blue, hypothetical proteins with unknown function are shown in yellow, and ORFs with no known homologies are shown in gray.

We also examined the CSF specimens to decipher whether there were viruses present in the CSF of all subjects studied. We created assemblies from the contigs of all study subjects using highly stringent criteria and examined those assemblies to decipher which study subjects contributed to each virus assembly. Of the 2429 CSF assemblies created, we found only 2 virus assemblies (0.08%) that were derived from all 20 subjects (Supplementary Figures S12A,B). The majority of the assemblies created (74.1%) were derived from 1 or 2 subjects, with only 1.1% of the assemblies derived from 10 or more subjects. One of the two viruses that were assembled from all 20 subjects include a putative 66.4 kb Myovirus that has restriction/modification enzymes and includes a site-specific integrase suggesting it has a lysogenic lifestyle (Figure 6A). The other includes a putative 37.0 kb Siphovirus that has a transposase that also suggests it has a lysogenic lifestyle (Figure 6B). Of the 27 virus assemblies created from 10 or more subjects, 13 (48.1%) had genes, including transposases/integrases/repressors that suggested lysogenic lifestyles. We could not identify the bacteria hosts of the viruses we assembled.

### Ion Torrent vs. Illumina Sequencing

We noted that the specimens belonging to each ecological cluster seemed to correlate with sequencing modality, where the urine, feces, and saliva cluster were all sequenced by semiconductor sequencing (Rothberg et al., 2011), while the plasma, CSF, body fluid, and milk cluster were all sequenced with Illumina technology (Bentley et al., 2008). To verify that sequence modality was not responsible for the ecological clusters, we sequenced the CSF specimens using both semiconductor and Illumina sequence technology and compared results. We characterized the alpha diversity present in these CSF specimens sequenced via semiconductor sequencing to decipher whether there were substantial differences observed based on the sequencing modality. The CSF specimens sequenced via semiconductor sequencing generally had slightly higher alpha diversity than was observed in these same specimens sequenced using Illumina, but remained in the same ecological cluster (Supplementary Figure S13). We also examined beta diversity via PCoA and tested significance using a pairwise Adonis test. Here, we found that the CSF specimens were not significantly different based on the sequencing modality used (Supplementary Figure S14). These data indicate that sequencing method was not responsible for the ecological clusters observed.

## Discussion

Prevailing clinical dogma suggests that the CNS and some other surfaces are generally sterile in healthy individuals. Here we prove otherwise using culture-independent methods to assess the microbial constituents of CSF samples to reveal a pervasive virome that appears to be partially shared with plasma, body fluids, and breast milk. Our discovery of a CSF virome suggests that the CNS is colonized by a somewhat diverse viral community and challenges the clinical dogma that the CNS is free of microbes in healthy individuals. While herpesviruses have been known for some time to inhabit the CNS of symptomatic individuals (Meyding-Lamade and Strank, 2012), the majority of viruses that could be identified were bacteriophages (Supplementary Figure S8). These phages could merely be bystanders that have arrived in the CSF without any clear role, but they could serve a more functional role as non-host-derived immunity against potential bacterial invaders, as has been hypothesized by others (Dabrowska et al., 2005; Duerkop and Hooper, 2013; Barr, 2017; Nguyen et al., 2017). Currently, there is limited evidence on whether these viral communities play any functional role in the CNS, but their discovery here obviates the need for further study in this field.

We recognize that contamination is of significant concern in identifying a virome present in a body site that previously was believed to be devoid of viruses in healthy people. The primary sources of any potential contamination in CSF samples would likely be derived from the skin, blood, or consumables used in the production of the viromes. We do not believe that the skin is a reasonable source of contamination because of the means by which the CSF is collected. The skin is first sterilized, and then a single puncture with a needle is used to collect the CSF. Because we examined the viromes only from Tube#3 (see Materials and Methods section for further details), it is highly unlikely that there could be sufficient remaining skin-derived viruses present from a single skin puncture after 4–8 mL of CSF have already been collected. Often, there is some blood in CSF specimens because blood vessels can sometimes be punctured as CSF is collected. However, for 7/20 of the subjects in this study, no blood was detected in their CSF (Supplementary Table S1). We noted no differences in the virome diversity or contents based on whether there were RBCs detected in the CSF. We tested water that had been taken through the exact same extraction process as the CSF to determine whether the presence of viruses could have been derived from the virome extraction process. While we could find viruses in the virome extracted sterile water, they were not the same viruses we observed in the CSF viromes, nor did these viromes have similar diversity (Figure 3).

We characterized the viromes of 7 different body surfaces and revealed two separate viral ecological groups dominated by phages with varying properties: a high diversity viral group that included feces, saliva, and urine, and a low diversity group made up of CSF, body fluids, plasma, and breast milk. The robust and diverse bacterial communities inhabiting feces, saliva, and urine might explain why these sample sites had high virome diversity. The low diversity viral group, including CSF, body fluids, and plasma, generally had few if any bacterial communities associated, which likely explains why few VLPs were observed and the low diversity. Interestingly, breast milk was associated with the low diversity cluster, but is also known to harbor relatively diverse bacterial communities (Hunt et al., 2011; Pannaraj et al., 2017), so the ecological clustering of the specimen types cannot be explained merely by the presence of bacteria. The breast milk virome has unique features amongst the specimens, including the predominance of myoviruses (Figure 1) and the differences in beta diversity (Figure 2). In further analysis of breast milk viromes, we note that these populations are quite uneven, with the majority of the breast milk virome sequence reads belonging to just a few viruses. For example, the most abundant virus in the breast milk viromes represented 27.8 ± 2.3% of the virome reads, and the top two viruses represented 41.9 ± 3.5% of the reads. This uneven population likely contributed substantially to the low virome diversity of the breast milk specimens compared to the high virome diversity observed in other specimen types where bacterial diversity was relatively high. We observed a similar phenomenon when examining alpha diversity in the feces using the Shannon Index compared to the Chao1 index (Supplementary Figure S9 and Figure 3). The lower alpha diversity measured using the Shannon index likely was due the fact that the Shannon index does not account for the many low abundance viruses found in the gut. Because the viromes in this study were MDA amplified, biases could have been introduced that affected alpha diversity, however, we did not identify such biases in our prior study where we validated the methodology (Santiago-Rodriguez et al., 2015b). Additionally, MDA amplification may introduce biases when examining differential abundances of the viruses present (Roux et al., 2016). It is important to note that for most analyses in this study we assign reads to virus contigs, but the abundances we evaluate are based on the number of these different virus contigs, not the proportion of reads assigned to the contigs.

There are a number of limitations to the methods we used to isolate and sequence viruses that have been revealed in a series of recent studies (Hunt et al., 2011; De Vlaminck et al., 2013; Shah et al., 2014; Wylie et al., 2014, 2015; Briese et al., 2015; Conceicao-Neto et al., 2015; Kleiner et al., 2015; Roux et al., 2016). Those methods include sequential filtration and Cesium chloride density gradient centrifugation. We still chose to isolate and sequence these viromes using these existing techniques so that they could be directly compared to our substantial existing library of specimens sequenced using the same techniques. By keeping the methodology constant for all 7 specimen types used, we reduced biases between studies that arise by altering the virus processing protocols, and the consistency of our study protocols allowed us to establish the ecological clusters. Despite these benefits, the limitations of the protocols include, underrepresentation of eukaryotic and enveloped viruses, no accounting for RNA viruses, overrepresentation of small single stranded DNA viruses (although this has not been apparent in our studies), inability to perform quantitative analysis, filtering out larger viruses, and underrepresentation of smaller viruses due to density gradient methods. Membership of CSF viromes may differ from this study if other existing virome processing methodologies are used.

Identifying the source of the CSF virome is of substantial importance. Prior to performing this study, we hypothesized that the GI tract would be the primary source of viromes throughout the body due to transcytosis of viruses across the gut epithelium into the bloodstream (Nguyen et al., 2017), where those viruses may then be distributed to the tissues and body fluids. Thus, body sites that are relatively devoid of bacteria would have their viromes established from the GI tract. We expected that CSF, body fluid, plasma, and perhaps even breast milk viromes would all have significant similarity to each other and contain viral types originating from the GI tract. Our data did suggest that CSF, body fluid, plasma and to an extent breast milk, all contained similar, low diversity viromes, but we could not establish a strong connection between these viromes and the GI tract. Instead, we identified greater similarity between CSF and salivary viromes than we did for fecal viromes (Figure 4). It is an intriguing possibility that on some body surfaces with few if any bacteria, transcytosis may allow viruses to access these compartments via the bloodstream. We hypothesized that the GI tract was a primary source of these viruses due to its large surface area, however, the oral cavity contains a large surface area and could provide the primary source for the low diversity virome seen in CSF samples (Collins and Dawes, 1987). We did not identify any strong evidence of the source of these viruses by examining the structures of the viruses that were present in multiple different subjects, but did find evidence that many of them had temperate lifestyles (Figure 6). Further studies are required to establish whether viruses may transcytose across oral mucosal layers and seed other body surfaces.

While we identified viruses in the CSF of this study population, we were not able to ascertain whether they were stable members of the CSF virome. Because this study was not longitudinal, we do not know whether the viruses we observed would be the same viruses we could identify if we examined the CSF virome of these same individuals days, weeks, or even months later. The invasive nature of obtaining CSF via lumbar puncture prohibited us from performing such an analysis. We believe that many of the bacteriophages found in the CSF arrived into the CNS via transcytosis and do not have viable host cells they are capable of infecting in the CSF. Thus, many of these viruses likely are transient members of the CSF virome.

The discovery of a virome in the CSF and various body fluids suggests that there are no body surfaces that are free of microbes. To date, viromes have been identified in saliva, dental plaque, feces, skin surfaces, breast milk, urine, lungs, blood, the vagina, and now body fluids and CSF. While the contents of viromes may appear different based on the techniques used to characterize them, each of the viromes detailed in this study were characterized using the same methodology. This methodology reveals taxonomic differences amongst viromes, including an abundance of microviruses in the feces, siphoviruses in the mouth, and myoviruses in breast milk. The most striking feature amongst the viromes was the high and low diversity ecological clusters identified, where body surfaces with many bacteria generally had high virome diversity, while others such as CSF and body fluids had low diversity. Even in the CSF and body fluid of infected individuals, there was low virome diversity. Thus, differences in alpha diversity appear to be features of each body surface. Further study is necessary to determine the origin of the viruses on many of these surfaces and to decipher why certain surfaces have low viral diversity compared to their counterparts.

## Ethics Statement

Human subject involvement in this study was approved by the University of California, San Diego Administrative Panel on Human Subjects in Medical Research. The study was certified as category 4 exempt, which does not require informed consent on behalf of the study subjects. The research was categorized as exempt because it involved the collection or study of existing data, documents, records, pathological specimens, or diagnostic specimens that were recorded in such a manner that the subjects could not be identified, directly or through identifiers linked to the subjects.

## Author Contributions

CG and DP conceived and designed experiments. MeL, JS, LS, KA, JC, and MaL performed the experiments. CG, JB, and DP analyzed the data. CG, JB, RS, and DP wrote and critically reviewed the manuscript.

## Conflict of Interest Statement

The authors declare that the research was conducted in the absence of any commercial or financial relationships that could be construed as a potential conflict of interest.

## Footnotes

1 www.thepridelaboratory.org

2 ftp://ftp.ncbi.nlm.nih.gov/genomes/genbank/bacteria/

3 www.thepridelaboratory.org

4 www.thepridelaboratory.org

## Supplementary Material

FIGURE S1 Epifluorescence microscopy of Virus-Like Particles (VLPs) present in the CSF **(A)** or in saliva for comparison **(B)**.

FIGURE S2 Size distribution of the contigs for body fluid and cerebrospinal fluid samples. Contig length is shown on the y-axis and the sample ID is shown on the x-axis. The break in the y-axis represents a change in the scale of axis intervals.

FIGURE S3 Percentages of virome reads (± standard error) belonging to contigs with significant sequence similarities within the NCBI NR database. The percentage of reads was determined based on the raw number of reads used to assemble each contig. The percentage of reads is shown on the y-axis, and the category of BLASTX homolog is shown on the x-axis.

FIGURE S4 Percentages of contigs with significant sequence similarities within the IMG/VR v2.0 database. Contigs with significant homologs in the database are shown in black and those with no significant homologs are shown in gray. The percentage of contigs is shown on the y-axis and the specimen type and number is shown on the x-axis.

FIGURE S5 Percentages of contigs with BLASTX homologs in the IMG/VR v2.0 database and the associated environments from which top homologs were derived. The percentage of contigs is shown on the y-axis and the specimen type and number is shown on the x-axis.

FIGURE S6 Representation of beta-diversity based on Bray Curtis distances, shown in Principal coordinates analysis of body fluids and cerebrospinal fluid. CSF specimens are represented by blue circles and body fluid specimens are represented by red circles. Ellipses are drawn at 95% confidence intervals for sample type.

FIGURE S7 Percentages of contigs (± standard error) with significant sequence similarities using TBLASTX analysis of the virus database at NCBI. The percentage of contigs is shown on the y-axis and the specimen type is shown on the x-axis.

FIGURE S8 Proportion of viral contigs with TBLASTX hits to the specified virus families. The y-axis represents the percentage of reads belonging to contigs homologous to each family, or that were unclassified dsDNA phages or other viruses. Fluid and CSF from subjects with known infections is demonstrated above the bars.

FIGURE S9 Homologous Virus Diversity Index rarefactions based on the Shannon Index of viral communities from various specimen types. The x-axis represents the number of virome reads sampled, and the y-axis represents virus abundance.

FIGURE S10 Diagram of contig 1516 assembled from saliva, stool, and urine viromes. The portions of the contig that were represented in the saliva of eight different subjects, in the stool of seven different subjects, and in the urine of four different subjects are shown below. Putative ORFs and their direction are indicated by the arrows at the top of the diagram. ORFs that had significant homologs (BLASTX E-score < 10^–5^) are indicated by the text above each arrow.

FIGURE S11 Diagram of contig 20 assembled from body fluid and CSF viromes. The portions of the contig that were represented in the body fluids of five different subjects and in the CSF of 20 different subjects are shown below. Putative ORFs and their direction are indicated by the arrows at the top of the diagram. ORFs that had significant homologs (BLASTX E-score < 10^–5^) are indicated by the text above each arrow.

FIGURE S12 Bar graphs representing the number of subjects that contributed to each assembled CSF contig **(A)** and the percentage of contigs constructed from the different numbers of subjects **(B)**. The x-axis represents the number of different subjects and the y-axis represents the number of contigs or the percentage of contigs constructed.

FIGURE S13 Homologous Virus Diversity Index rarefactions based on the Chao1 Index of viral communities from various specimen types. The x-axis represents the number of virome reads sampled, and the y-axis represents homologous diversity. The CSF specimens sequenced using semiconductor sequencing (yellow circles) had higher diversity than those sequenced by Illumina (white circles), but remained in the low diversity cluster.

FIGURE S14 Representation of beta-diversity based on Bray Curtis distances, shown in Principal coordinates analysis of the viral communities. The CSF specimens sequenced using semiconductor sequencing (pink circles) clustered similarly to those sequenced by Illumina (blue circles). Ellipses are drawn at 95% confidence intervals for sample type.

TABLE S1 Characteristics of study subjects/specimens.

TABLE S2 Contig metrics from study specimens.

TABLE S3 Contig metrics from CSF specimens.

TABLE S4 IMG/VR BLASTX homolog habitats.
