# Supplementary figures and images for: The Virome of Cerebrospinal Fluid: Viruses Where We Once Thought There Were None
Source: Front Microbiol. 2019 Sep 6;10:2061. doi: 10.3389/fmicb.2019.02061 (PMC6742758; doi:10.3389/fmicb.2019.02061)

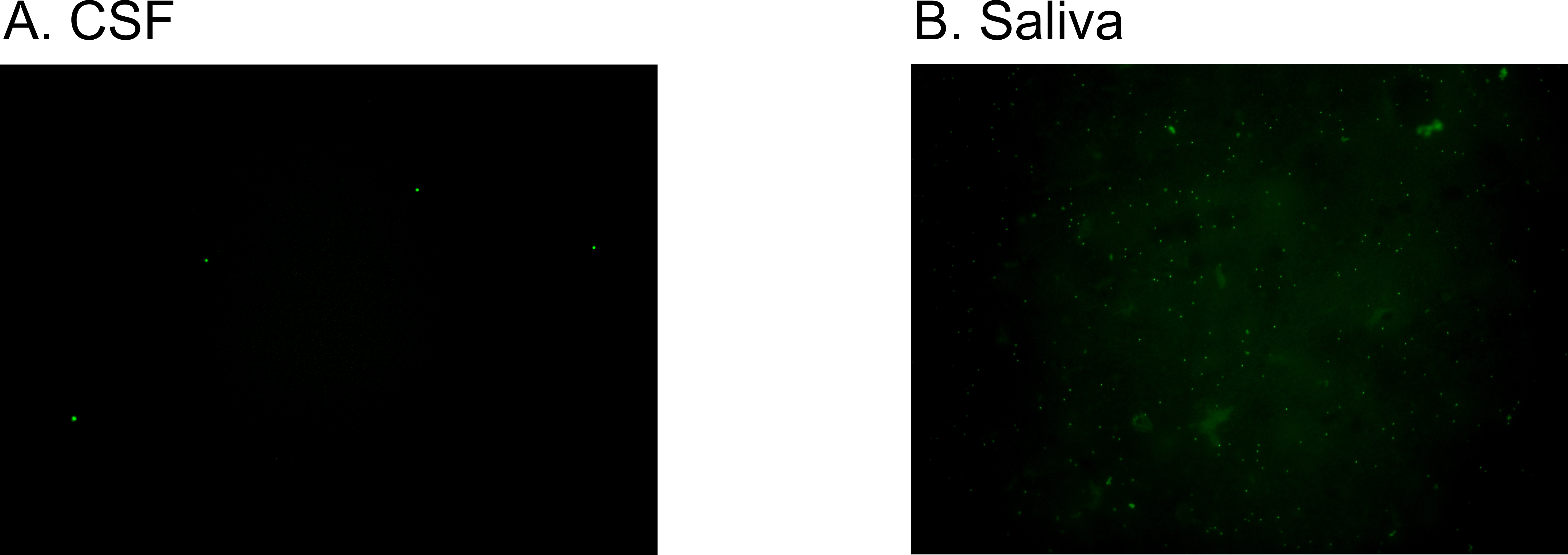

Supplement: FIGURE S1 — Epifluorescence microscopy of Virus-Like Particles (VLPs) present in the CSF (A) or in saliva for comparison (B). [file Image_1.TIF]

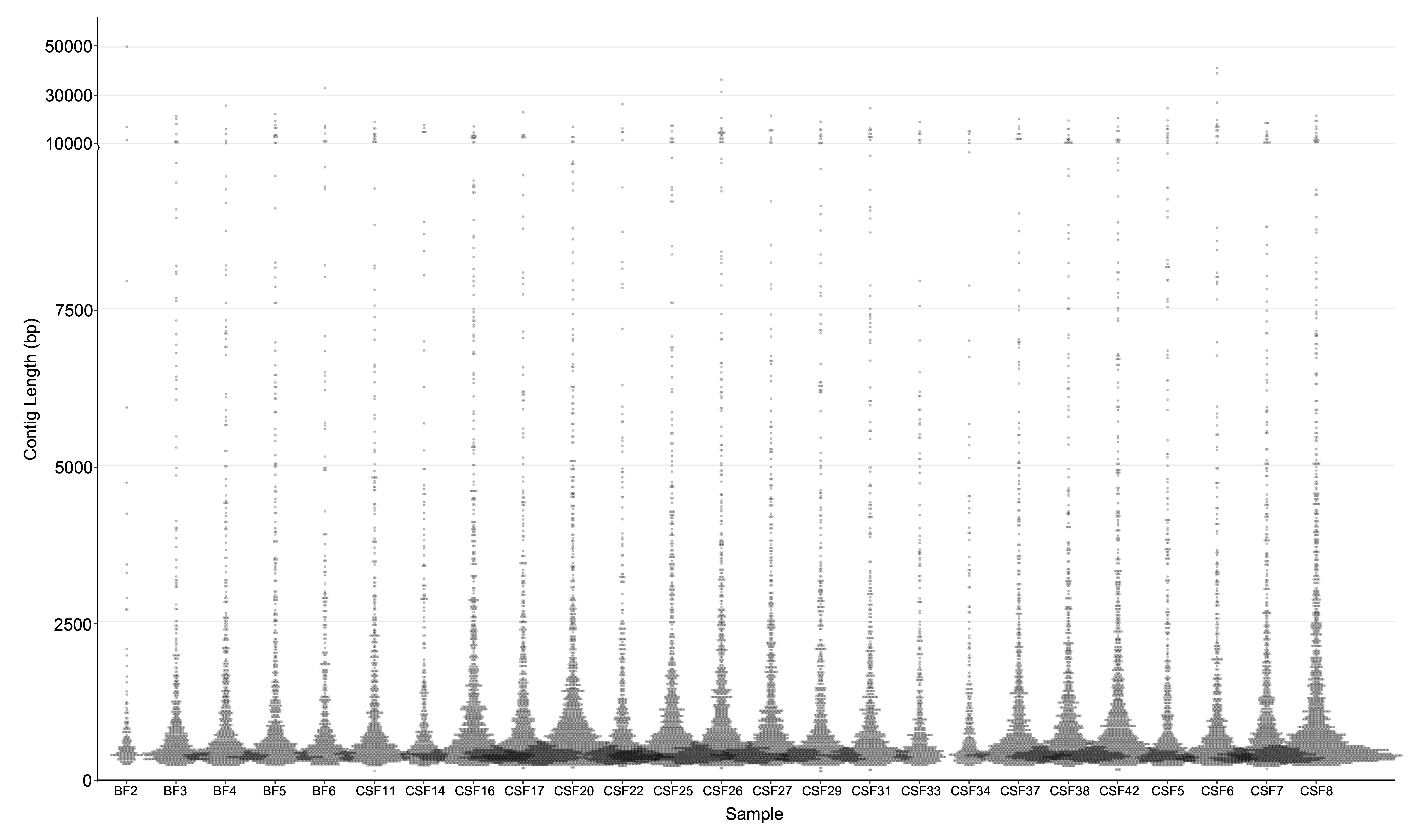

Supplement: FIGURE S2 — Size distribution of the contigs for body fluid and cerebrospinal fluid samples. Contig length is shown on the y-axis and the sample ID is shown on the x-axis. The break in the y-axis represents a change in the scale of axis intervals. [file Image_2.TIFF]

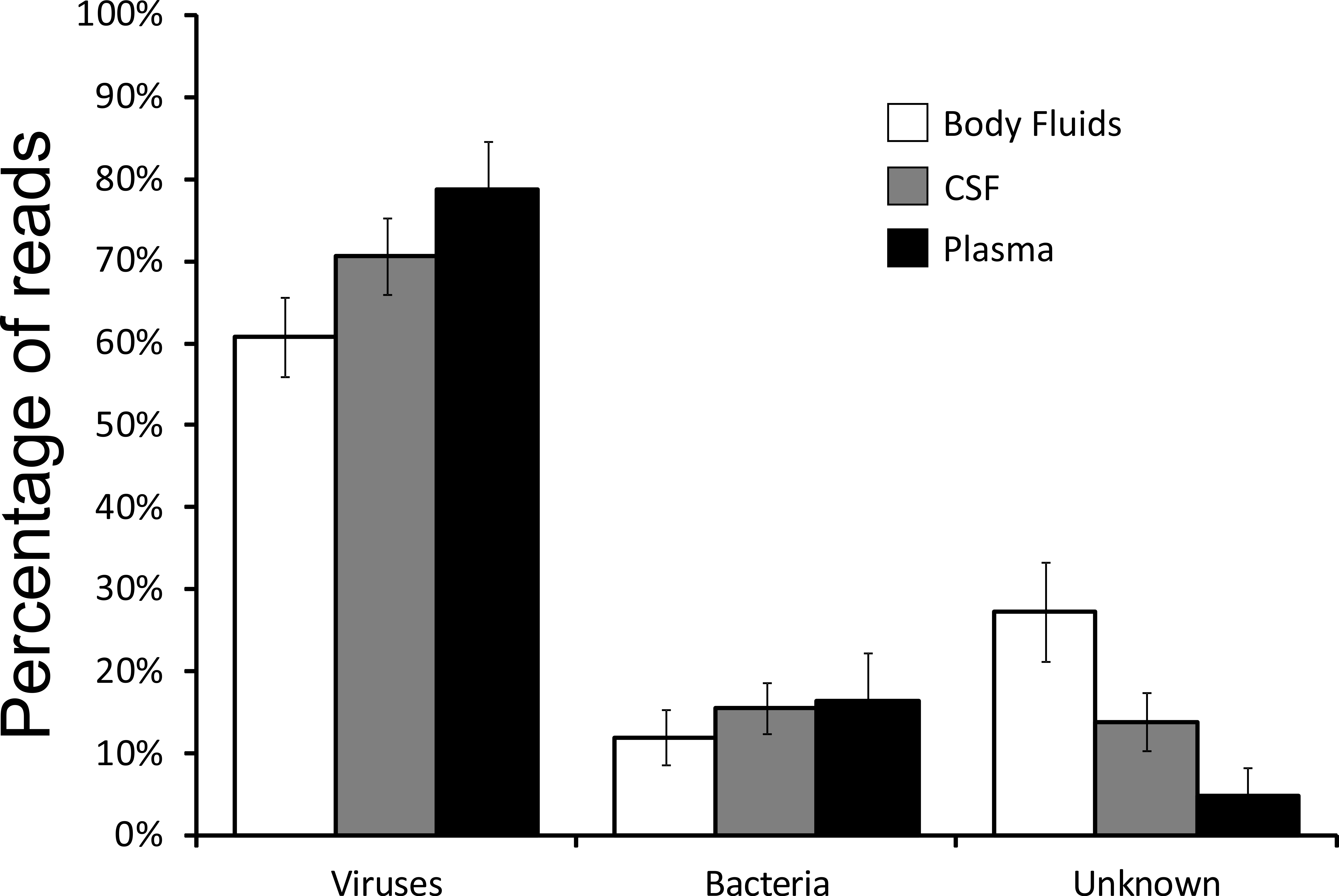

Supplement: FIGURE S3 — Percentages of virome reads (± standard error) belonging to contigs with significant sequence similarities within the NCBI NR database. The percentage of reads was determined based on the raw number of reads used to assemble each contig. The percentage of reads is shown on the y-axis, and the category of BLASTX homolog is shown on the x-axis. [file Image_3.TIF]

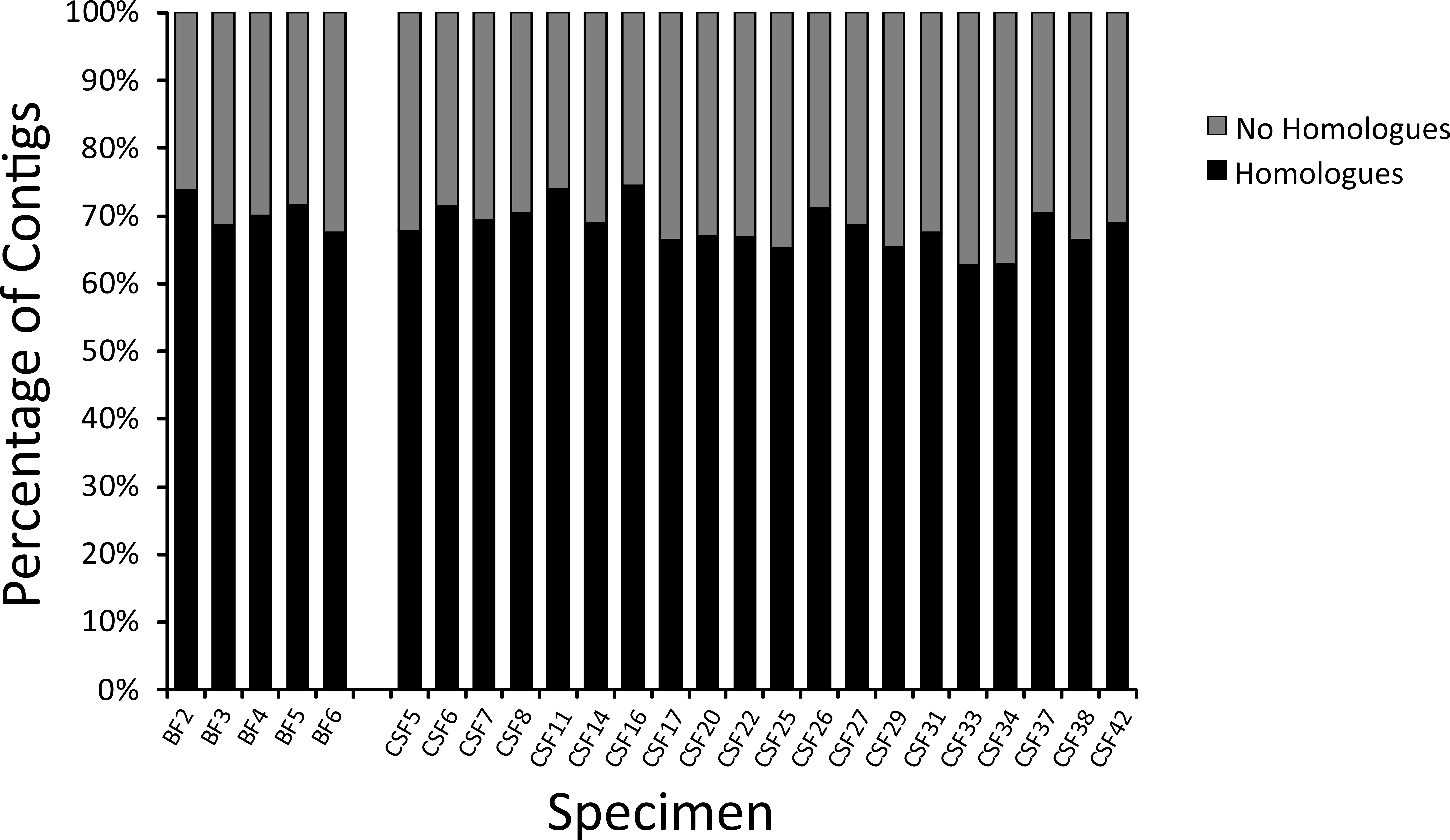

Supplement: FIGURE S4 — Percentages of contigs with significant sequence similarities within the IMG/VR v2.0 database. Contigs with significant homologs in the database are shown in black and those with no significant homologs are shown in gray. The percentage of contigs is shown on the y-axis and the specimen type and number is shown on the x-axis. [file Image_4.TIF]

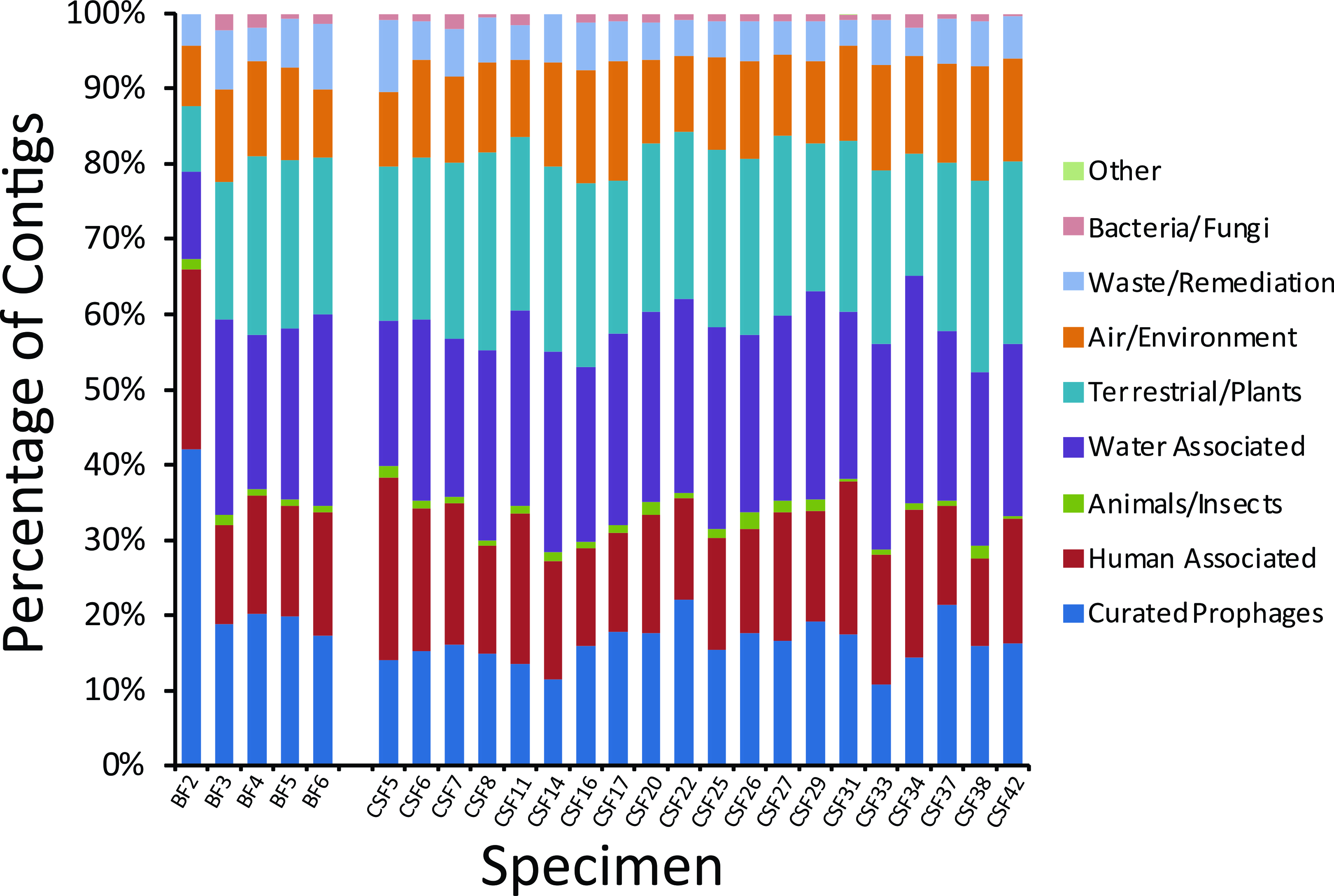

Supplement: FIGURE S5 — Percentages of contigs with BLASTX homologs in the IMG/VR v2.0 database and the associated environments from which top homologs were derived. The percentage of contigs is shown on the y-axis and the specimen type and number is shown on the x-axis. [file Image_5.JPEG]

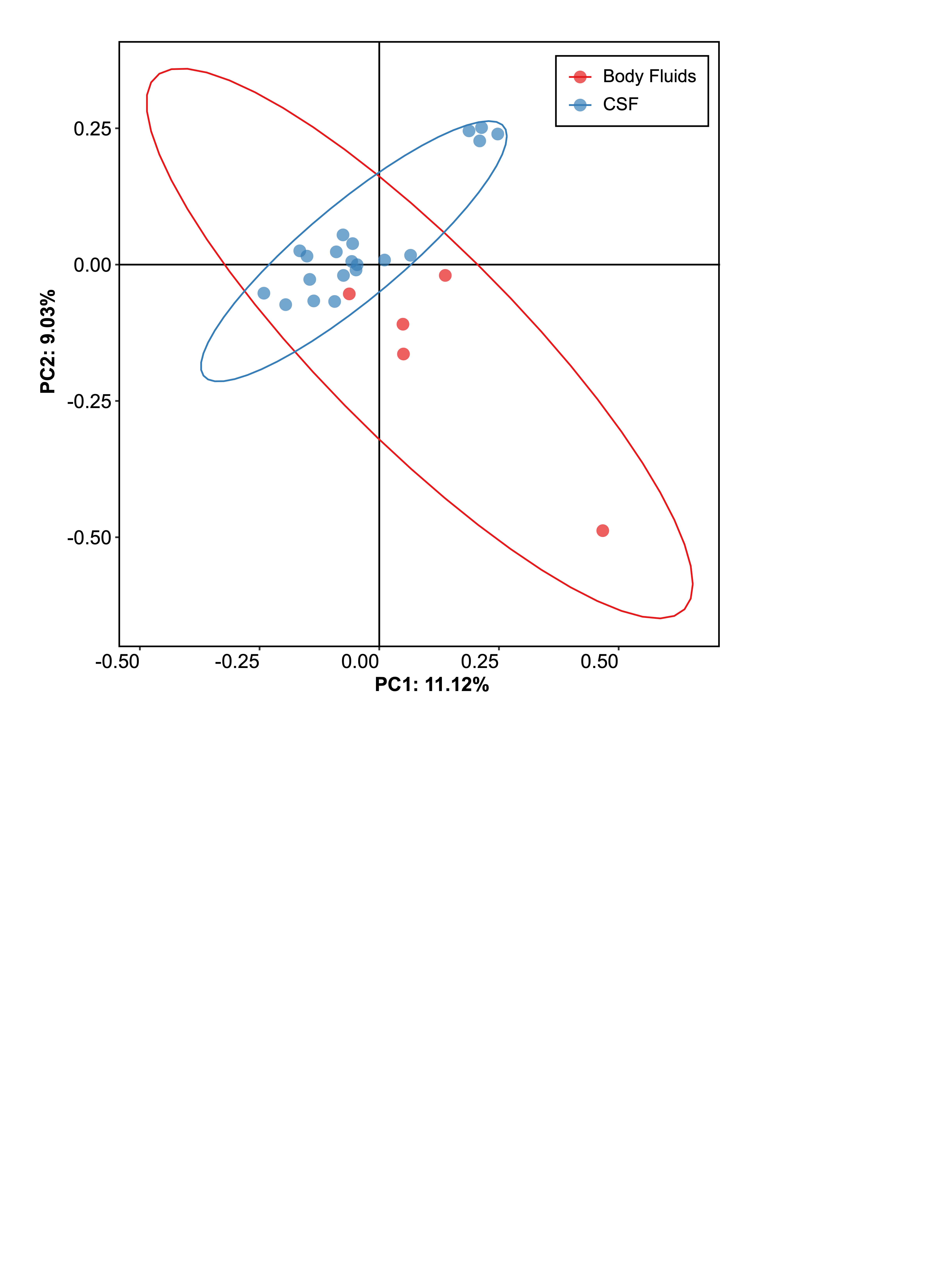

Supplement: FIGURE S6 — Representation of beta-diversity based on Bray Curtis distances, shown in Principal coordinates analysis of body fluids and cerebrospinal fluid. CSF specimens are represented by blue circles and body fluid specimens are represented by red circles. Ellipses are drawn at 95% confidence intervals for sample type. [file Image_6.TIFF]

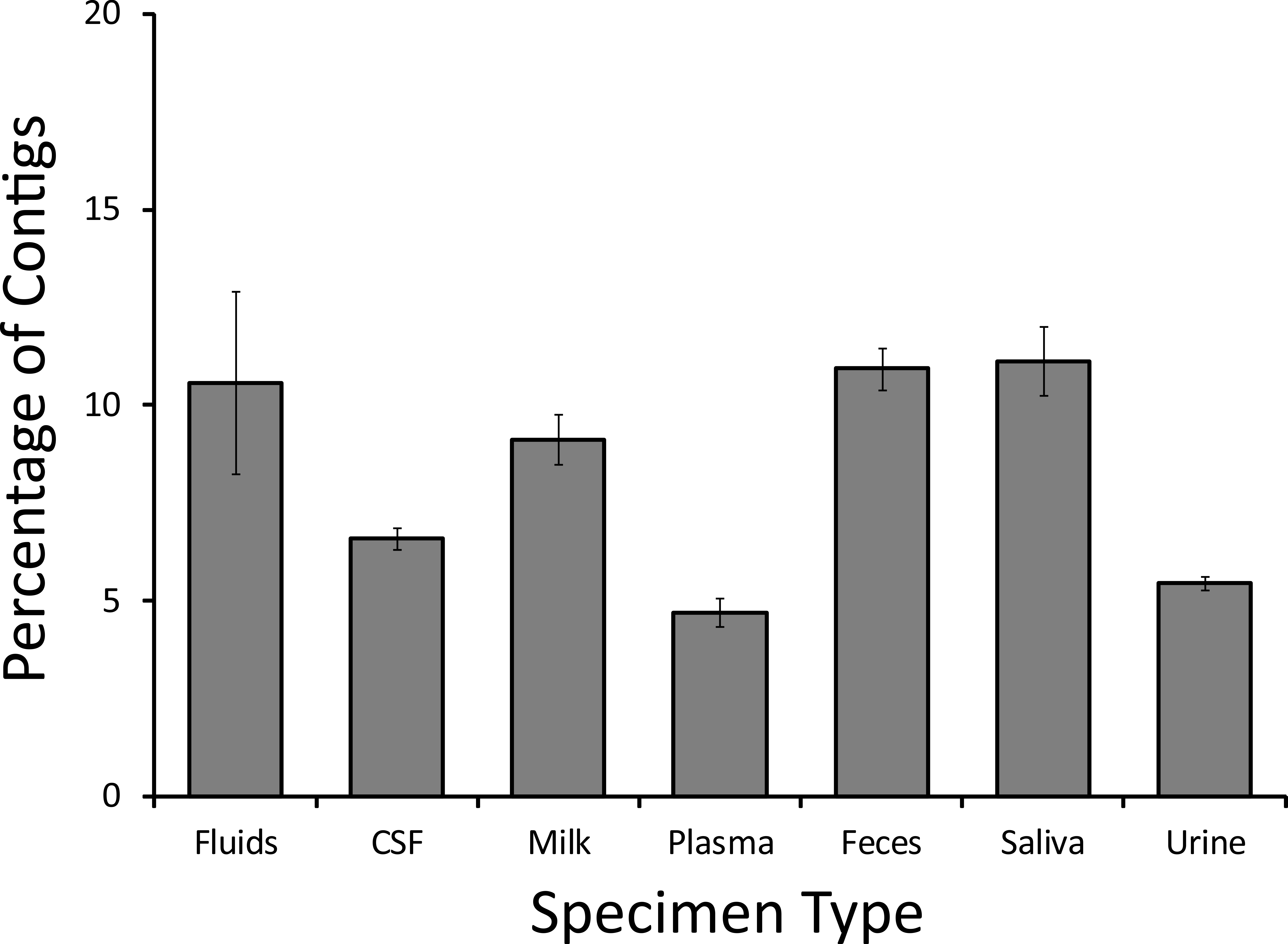

Supplement: FIGURE S7 — Percentages of contigs (± standard error) with significant sequence similarities using TBLASTX analysis of the virus database at NCBI. The percentage of contigs is shown on the y-axis and the specimen type is shown on the x-axis. [file Image_7.TIF]

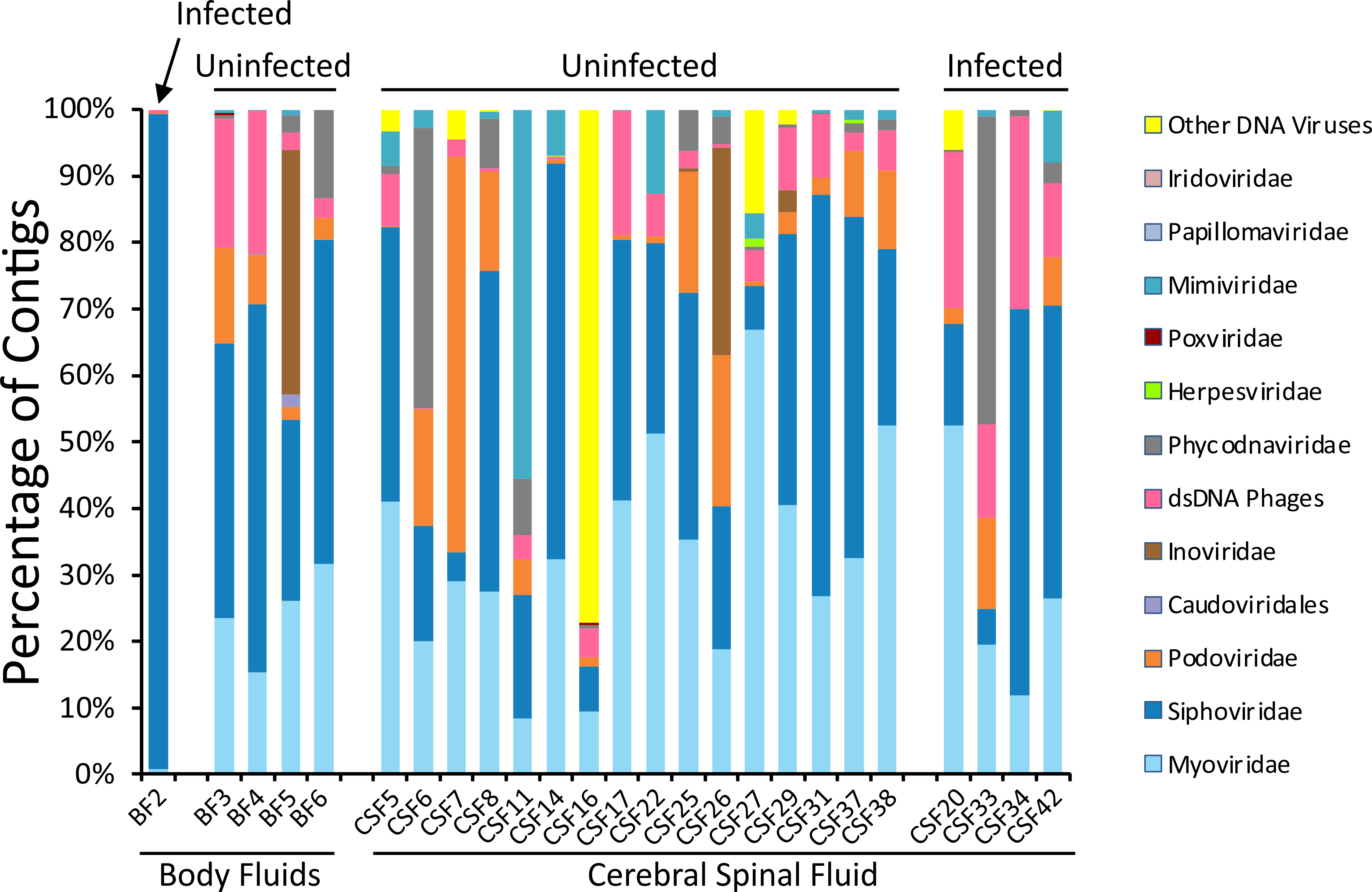

Supplement: FIGURE S8 — Proportion of viral contigs with TBLASTX hits to the specified virus families. The y-axis represents the percentage of reads belonging to contigs homologous to each family, or that were unclassified dsDNA phages or other viruses. Fluid and CSF from subjects with known infections is demonstrated above the bars. [file Image_8.TIF]

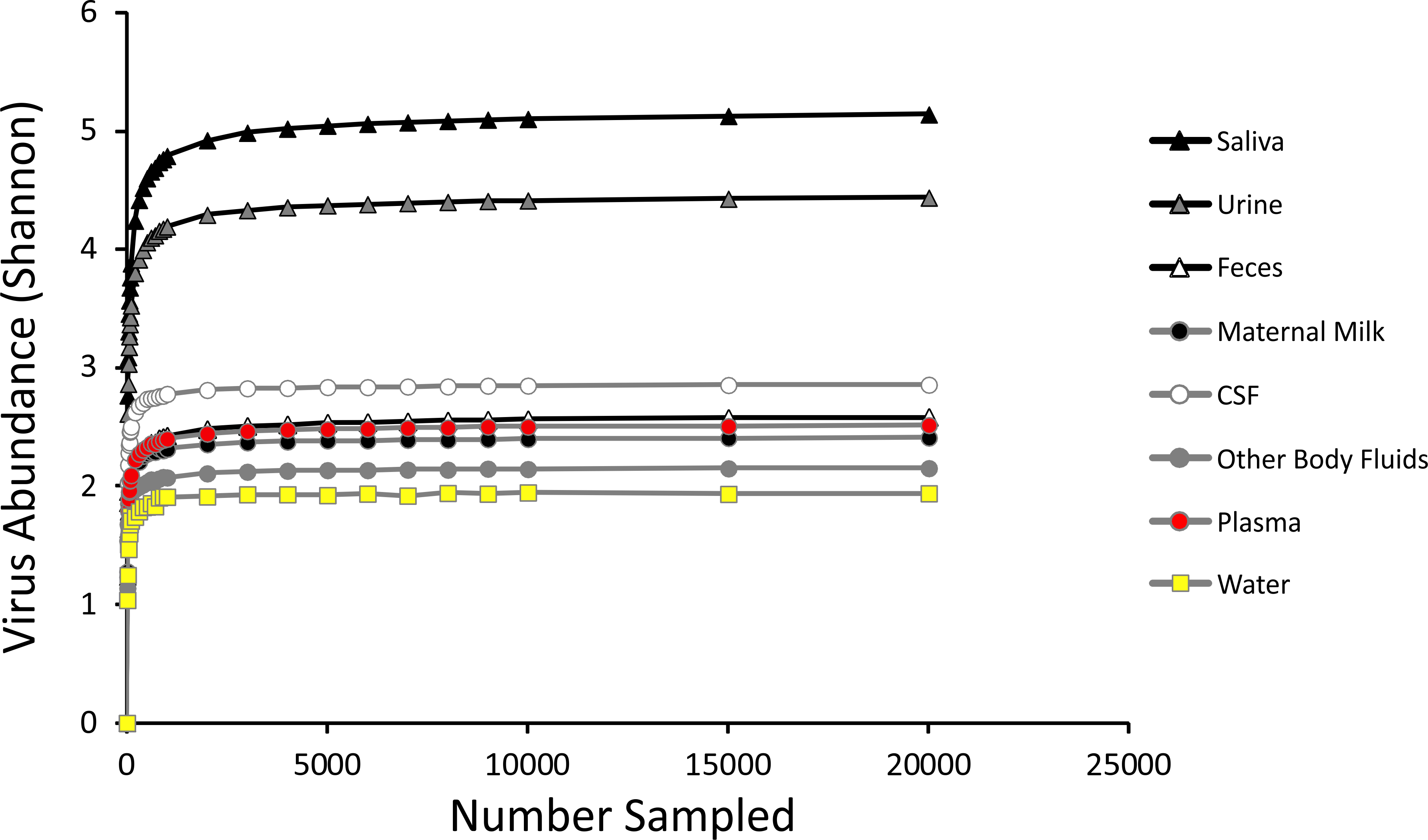

Supplement: FIGURE S9 — Homologous Virus Diversity Index rarefactions based on the Shannon Index of viral communities from various specimen types. The x-axis represents the number of virome reads sampled, and the y-axis represents virus abundance. [file Image_9.TIF]

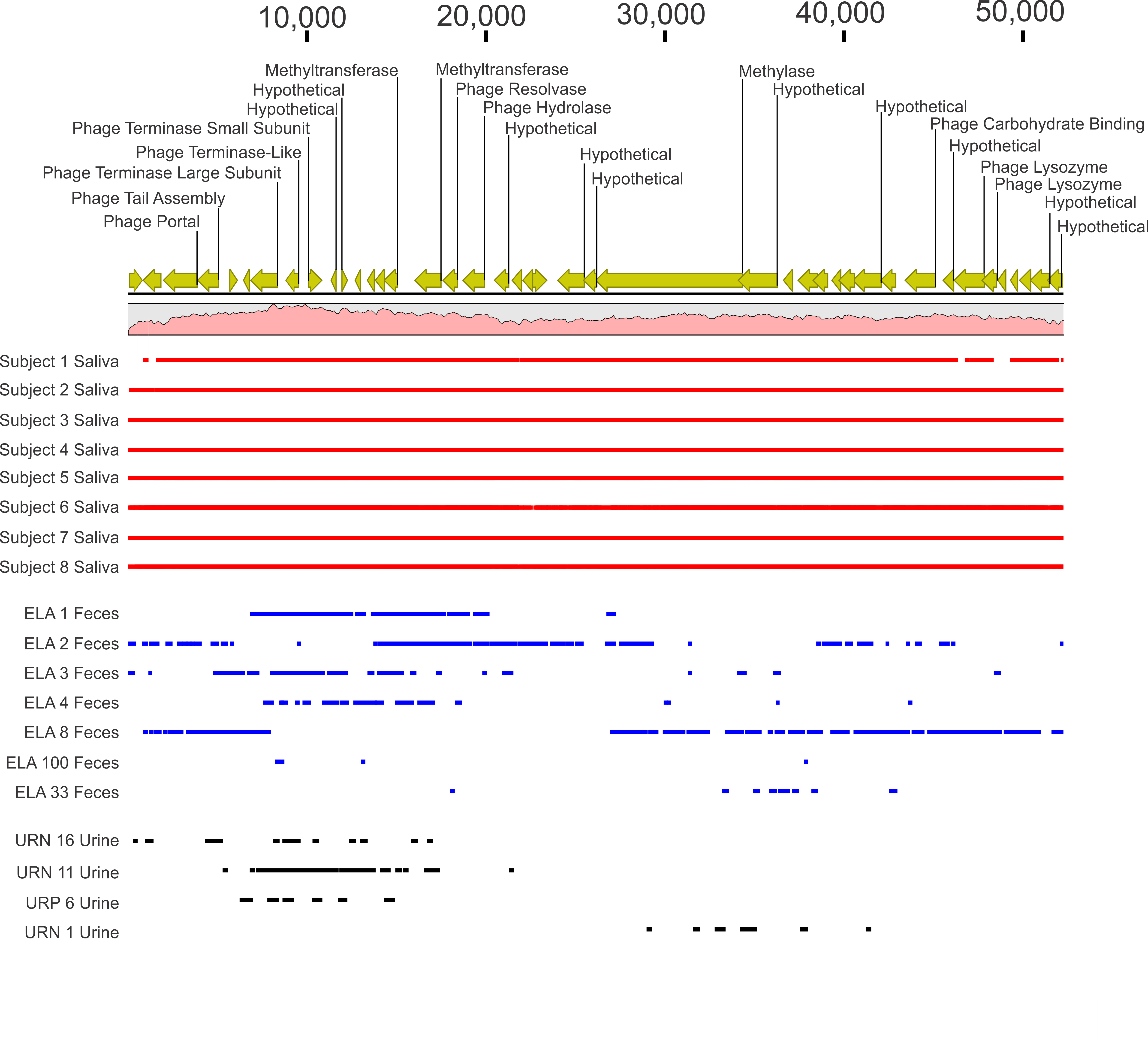

Supplement: FIGURE S10 — Diagram of contig 1516 assembled from saliva, stool, and urine viromes. The portions of the contig that were represented in the saliva of eight different subjects, in the stool of seven different subjects, and in the urine of four different subjects are shown below. Putative ORFs and their direction are indicated by the arrows at the top of the diagram. ORFs that had significant homologs (BLASTX E-score < 10–5) are indicated by the text above each arrow. [file Image_10.TIF]

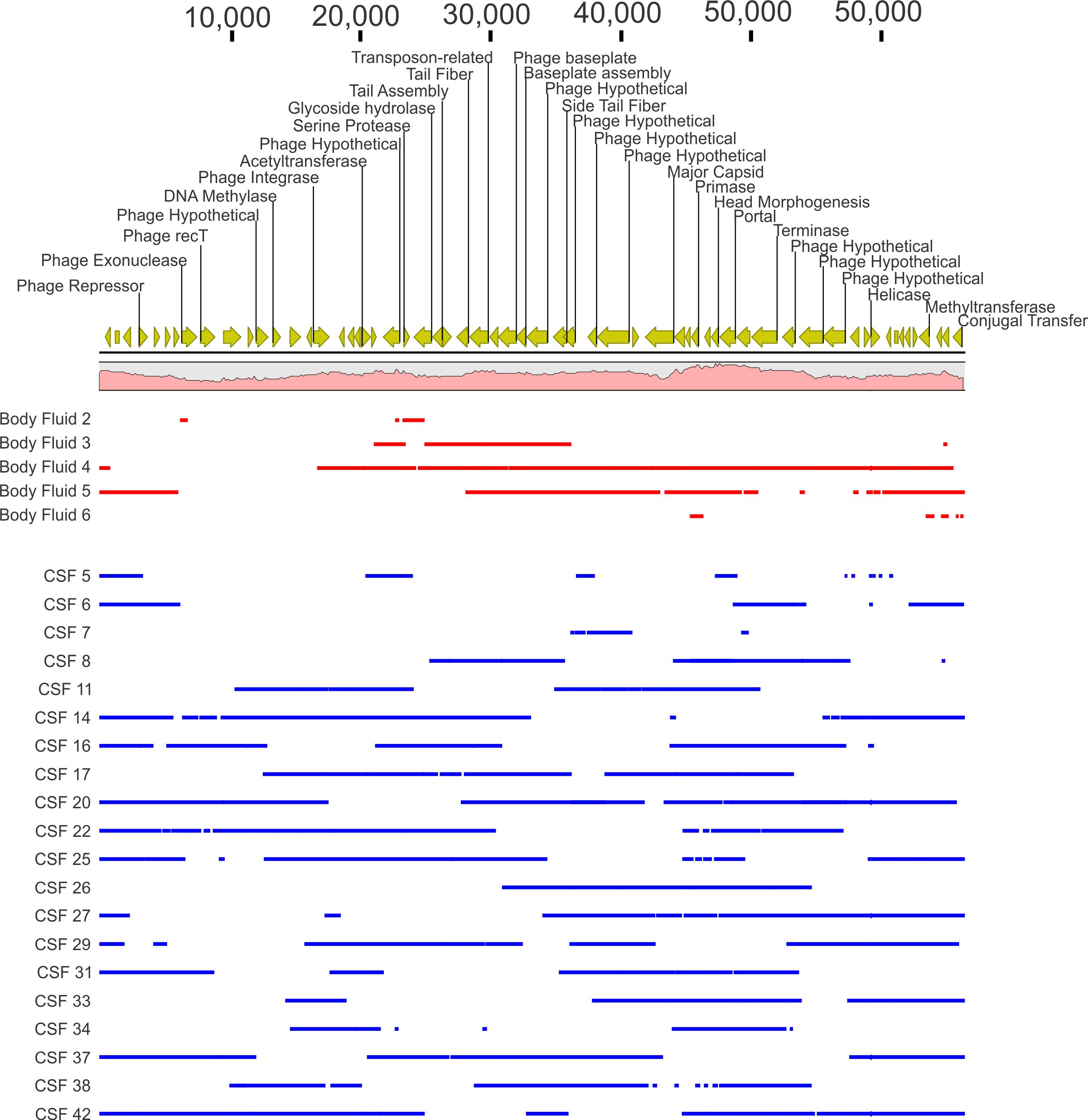

Supplement: FIGURE S11 — Diagram of contig 20 assembled from body fluid and CSF viromes. The portions of the contig that were represented in the body fluids of five different subjects and in the CSF of 20 different subjects are shown below. Putative ORFs and their direction are indicated by the arrows at the top of the diagram. ORFs that had significant homologs (BLASTX E-score < 10–5) are indicated by the text above each arrow. [file Image_11.JPEG]

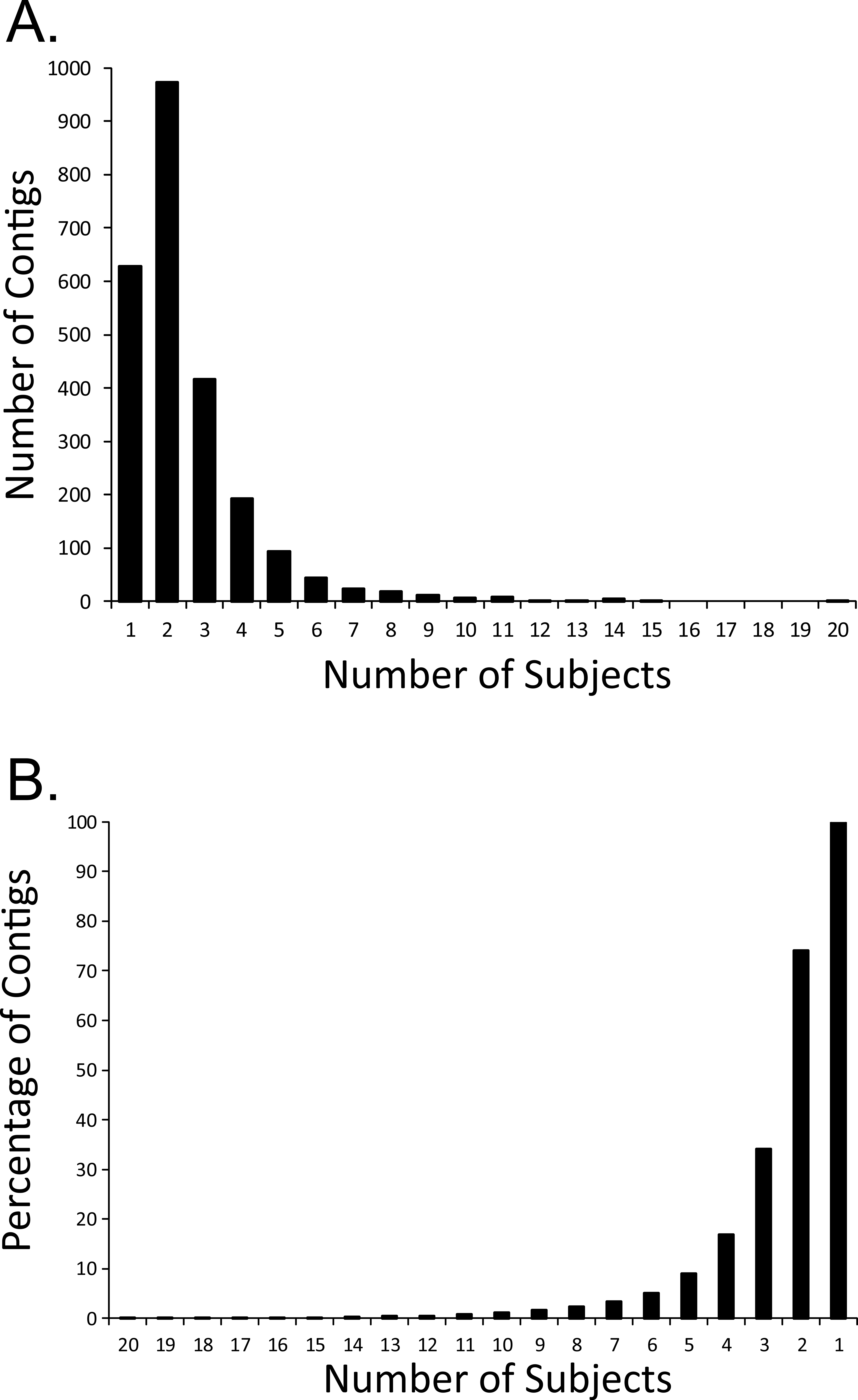

Supplement: FIGURE S12 — Bar graphs representing the number of subjects that contributed to each assembled CSF contig (A) and the percentage of contigs constructed from the different numbers of subjects (B). The x-axis represents the number of different subjects and the y-axis represents the number of contigs or the percentage of contigs constructed. [file Image_12.TIF]

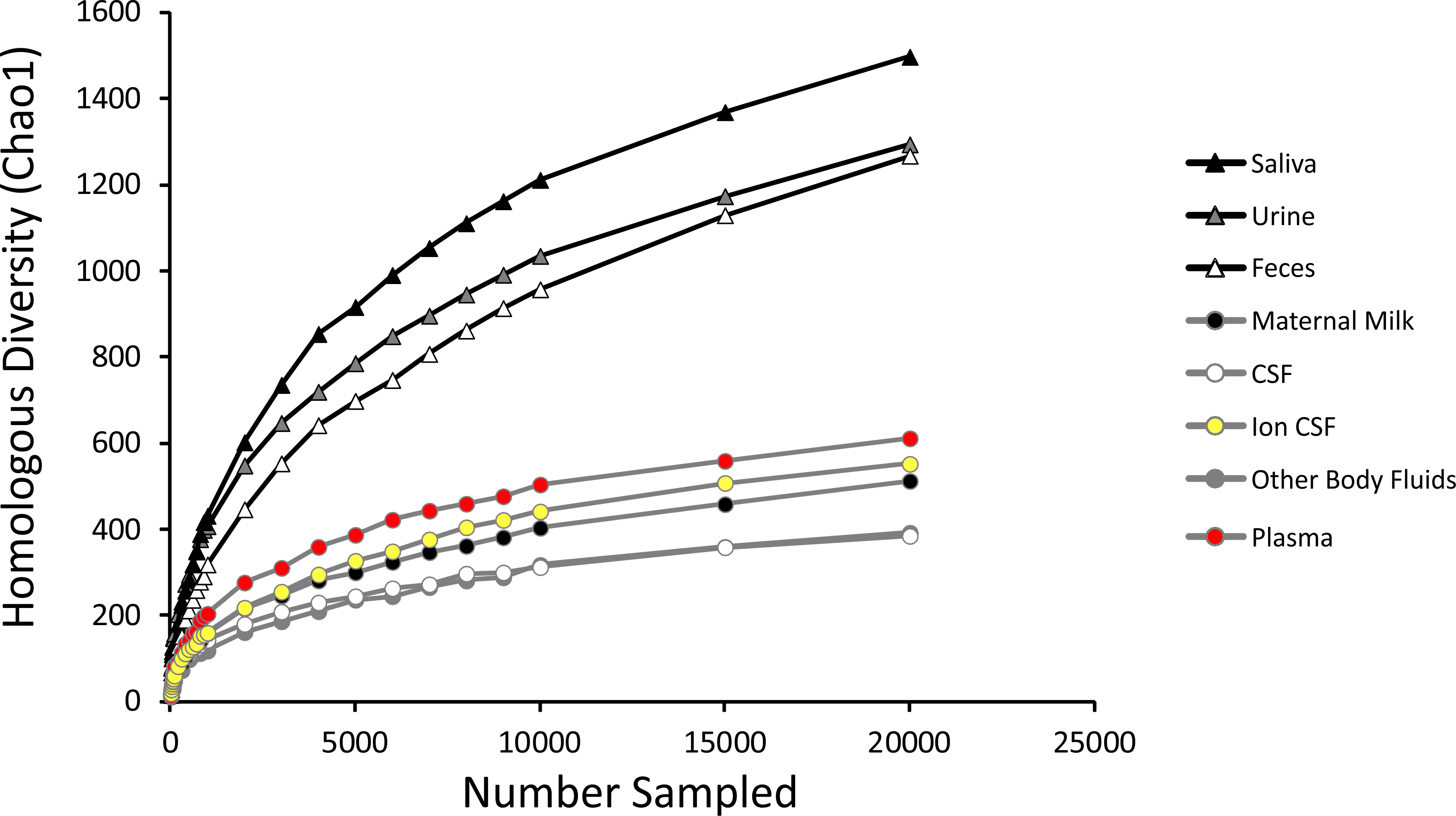

Supplement: FIGURE S13 — Homologous Virus Diversity Index rarefactions based on the Chao1 Index of viral communities from various specimen types. The x-axis represents the number of virome reads sampled, and the y-axis represents homologous diversity. The CSF specimens sequenced using semiconductor sequencing (yellow circles) had higher diversity than those sequenced by Illumina (white circles), but remained in the low diversity cluster. [file Image_13.TIF]

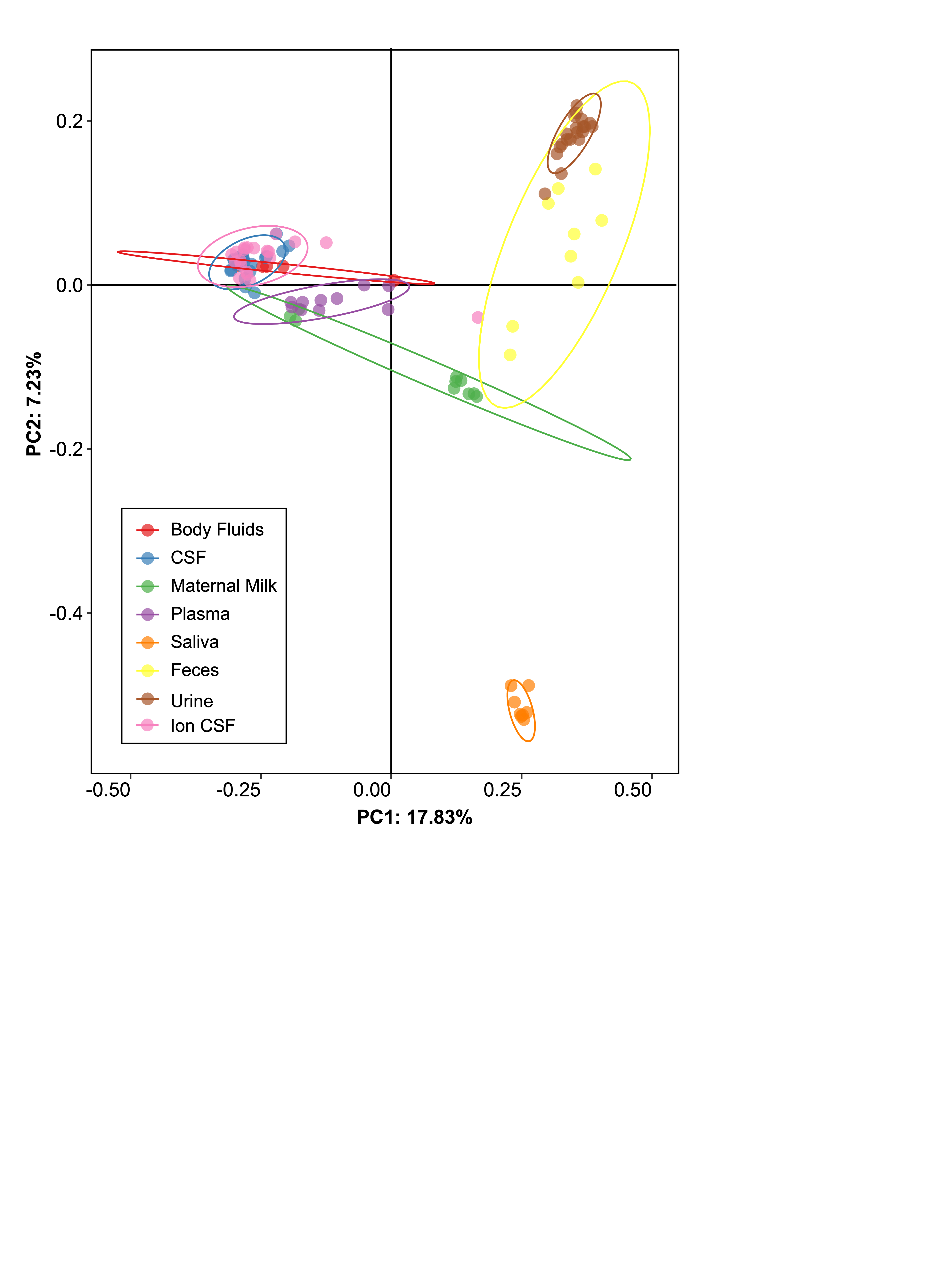

Supplement: FIGURE S14 — Representation of beta-diversity based on Bray Curtis distances, shown in Principal coordinates analysis of the viral communities. The CSF specimens sequenced using semiconductor sequencing (pink circles) clustered similarly to those sequenced by Illumina (blue circles). Ellipses are drawn at 95% confidence intervals for sample type. [file Image_14.JPEG]
