# Supplementary material for: The Virome of Cerebrospinal Fluid: Viruses Where We Once Thought There Were None
Source: Front Microbiol. 2019 Sep 6;10:2061. doi: 10.3389/fmicb.2019.02061 (PMC6742758; doi:10.3389/fmicb.2019.02061)
Supplement: TABLE S1 — Characteristics of study subjects/specimens. [file Data_Sheet_1.PDF]

Table S1. Characteristics of study subjects/specimens.

Subject	Age	Sex	WBC Count	RBC Count[b]	Fluid Type	Organism recovered	Comorbidities
Body fluids							
**BF2**[a]	**72**	**F**	**132**	**23,000**	**Bile fluid**	**Ecoli, Efaecium, Efaecalis**	**Cholangitis**
BF3	69	M	0	84	Hip fluid	None	R THR
BF4	57	M	255	49,000	Peritoneal fluid	None	Liver transplant, ascites
BF5	57	M	196	10,000	Peritoneal fluid	None	Alcohol cirrhosis
BF6	57	M	48	4,322	Peritoneal fluid	None	NASH cirrhosis
Cerebrospinal fluid							
CSF5	53	F	2	**1200**	CSF	None	Metastatic cancer
CSF6	34	F	1	0	CSF	None	Nystagnus
CSF7	45	M	12	230	CSF	None	Orbital fracture
CSF8	83	M	2	1250	CSF	None	Dementia
CSF11	20	F	0	12	CSF	None	Pseudotumor cerebri
CSF14	38	M	0	29	CSF	None	Lymphoma
CSF16	80	F	18	363	CSF	None	Chronic leukocytosis
CSF17	50	M	2	0	CSF	None	Schizophrenia
**CSF20**	**70**	**M**	**9**	**122**	**CSF**	**Mycobacterium fortuitum**	**VP shunt**
CSF22	63	M	4	38	CSF	None	Syphilis
CSF25	55	F	2	163	CSF	None	Pituitary adenoma
CSF26	48	M	2	0	CSF	None	Lymphoma
CSF27	48	F	0	16	CSF	None	Acute lymphocytic leukemia
CSF29	70	M	0	0	CSF	None	Acute myeloid leukemia
CSF31	19	M	6	0	CSF	None	Schizophrenia
**CSF33**	**43**	**F**	**36**	**70**	**CSF**	**Enterobacter cloacae**	**Meningitis**
**CSF34**	**32**	**M**	**63**	**0**	**CSF**	**Coccidioimycosis immitis**	**Meningitis**
**CSF37**	**61**	**M**	**16**	**151**	**CSF**	**None**	**Viral encephalitis**
CSF38	50	M	1	21	CSF	None	Oropharyngeal cancer
CSF42	46	F	4	0	CSF	None	Seizure, epilepsy
Plasma							
PL101		M	28	NA	Plasma	None	Liver transplant
PL102		M	53	NA	Plasma	None	Renal transplant

PL103	M	36	NA	Plasma	None	Renal transplant
PL104	M	67	NA	Plasma	None	Congestive heart failure
PL105	M	30	NA	Plasma	None	Renal transplant
PL106	M	57	NA	Plasma	None	Diabetes, pneumonia, osteomyelitis
PL107	M	51	NA	Plasma	None	Renal transplant
PL108	M	60	NA	Plasma	None	Chronic lymphocytic leukemia
PL109B	F	62	NA	Plasma	None	Amyloidosis, renal transplant
PL110A	M	30	NA	Plasma	None	Lymphoma

[a]Specimens in bold represent subjects diagnosed with clinical infections in body fluids or CSF.

[b]Not applicable.
