# Supplementary material for: The Virome of Cerebrospinal Fluid: Viruses Where We Once Thought There Were None
Source: Front Microbiol. 2019 Sep 6;10:2061. doi: 10.3389/fmicb.2019.02061 (PMC6742758; doi:10.3389/fmicb.2019.02061)
Supplement: TABLE S2 — Contig metrics from study specimens. [file Data_Sheet_2.pdf]

Table S2: Contig metrics from study specimens

	Reads	Contigs	Mean Length	Max Length	N50	Percent In Contigs[a]
Body fluids						
BF2	1,599,812	187	1,135	50,123	3,280	99.2%
BF3	549,642	653	1,106	21,402	2,040	91.9%
BF4	2,078,202	800	1,126	25,637	2,236	96.7%
BF5	833,562	807	1,230	22,142	2,836	96.4%
BF6	533,220	625	1,130	33,048	2,411	96.9%
Cerebrospinal fluid						
CSF5	3,937,012	200	3,692	24,512	4,992	97.4%
CSF6	2,036,270	209	3,732	41,235	5,239	97.0%
CSF7	3,210,150	306	3,131	18,757	4,174	75.9%
CSF8	2,284,002	1,942	1,190	21,506	2,426	87.0%
CSF11	3,434,702	838	1,210	18,847	2,370	96.3%
CSF14	1,381,272	113	3,293	17,681	4,138	86.3%
CSF16	2,820,892	1,327	1,234	17,063	2,455	96.6%
CSF17	1,014,736	251	2,862	22,913	3,448	95.0%
CSF20	1,871,550	417	2,703	16,895	3,384	78.9%
CSF22	2,374,046	168	2,987	26,239	3,743	92.5%
CSF25	1,500,202	1,395	1,025	17,680	1,712	96.4%
CSF26	2,099,950	390	3,198	36,466	4,060	95.1%
CSF27	2,021,242	1,210	1,095	15,755	1,961	98.2%
CSF29	1,087,248	239	3,216	18,964	4,187	94.0%
CSF31	2,613,976	225	3,284	24,562	4,601	94.1%
CSF33	1,452,374	133	3,273	18,858	4,359	96.6%
CSF34	628,986	86	3,178	15,067	4,235	94.0%
CSF37	1,053,472	1,054	1,114	20,197	2,062	97.7%
CSF38	1,075,856	1,241	1,089	17,436	1,978	88.2%
CSF42	3,204,546	1,463	1,099	20,488	2,026	88.9%
Plasma						
PL101	1,302,136	110	2,789	16,468	3,248	91.3%
PL102	820,614	216	2,714	10,100	3,088	98.3%

	PL103	199,598	181	2,339	9,416	2,754	93.9%
	PL104	1,165,144	175	2,735	12,977	3,500	98.0%
	PL105	1,571,212	278	2,801	15,312	3,433	98.7%
	PL106	1,381,986	245	2,726	25,293	3,405	97.8%
	PL107	964,986	314	3,123	48,877	3,975	98.3%
	PL108	472,864	172	2,495	10,847	2,678	98.0%
	PL109B	1,368,514	151	4,011	48,449	7,418	99.0%
	PL110A	1,447,334	360	3,057	13,733	3,057	97.7%
Water Controls							
	Water 1	1,414,660	211	2,826	40,392	4,166	94.4%
	Water 2	2,395,396	127	3,191	22,204	4,316	86.1%
	Water 3	2,050,120	148	3,514	26,044	5,006	94.7%

[a]Percentage of reads that were assembled into contigs
