# Supplementary material for: The Virome of Cerebrospinal Fluid: Viruses Where We Once Thought There Were None
Source: Front Microbiol. 2019 Sep 6;10:2061. doi: 10.3389/fmicb.2019.02061 (PMC6742758; doi:10.3389/fmicb.2019.02061)
Supplement: TABLE S3 — Contig metrics from CSF specimens. [file Data_Sheet_3.PDF]

Table S3: Contig metrics from CSF specimens

	Contigs 50[a]	Contigs 80[b]	Mean Length 50	Mean Length 80	Max Length 50	Max Length 80	N50 50	N50 80
Body fluid								
BF2	187	187	1,135	1,132	50,123	50,122	3,280	3,280
BF3	653	651	1,106	1,108	21,402	21,283	2,040	2,161
BF4	800	798	1,126	1,126	25,637	25,637	2,236	2,223
BF5	807	808	1,230	1,225	22,142	22,142	2,836	2,855
BF6	625	628	1,130	1,121	33,049	33,060	2,411	2,400
Cerebrospinal fluid								
CSF5	200	197	3,692	3,741	24,512	24,512	4,992	5,123
CSF6	209	210	3,732	3,712	41,235	41,235	5,239	5,239
CSF7	306	308	3,131	3,111	18,757	18,757	4,174	4,126
CSF8	1,942	1,948	1,190	1,184	21,506	21,492	2,426	2,431
CSF11	838	838	1,210	1,213	18,847	18,847	2,370	2,370
CSF14	113	114	3,293	3,263	17,681	17,681	4,138	4,109
CSF16	1,327	1,327	1,234	1,232	17,063	17,063	2,455	2,494
CSF17	251	251	2,862	2,860	22,913	22,913	3,448	3,444
CSF20	417	419	2,703	2,693	16,895	16,895	3,384	3,386
CSF22	168	167	2,987	3,005	26,239	26,239	3,743	3,814
CSF25	1,395	1,395	1,025	1,023	17,680	17,680	1,712	1,719
CSF26	390	388	3,198	3,212	36,466	36,466	4,060	4,113
CSF27	1,210	1,208	1,095	1,094	15,755	15,754	1,961	1,957
CSF29	239	239	3,216	3,216	18,964	18,964	4,187	4,200
CSF31	225	227	3,284	3,250	24,562	24,562	4,601	4,550
CSF33	133	133	3,273	3,272	18,858	18,858	4,359	4,708
CSF34	86	86	3,178	3,172	15,067	15,067	4,235	4,235
CSF37	1,054	1,056	1,114	1,109	20,197	20,197	2,062	2,084
CSF38	1,241	1,239	1,089	1,086	17,436	17,436	1,978	1,983
CSF42	1,463	1,467	1,099	1,097	20,488	20,488	2,026	2,039

[a]Contigs constructed from virome reads at 98% identity with a minimum of 50% read overlap

[b]Contigs constructed from virome reads at 98% identity with a minimum of 80% read overlap
