# Supplementary material for: The Virome of Cerebrospinal Fluid: Viruses Where We Once Thought There Were None
Source: Front Microbiol. 2019 Sep 6;10:2061. doi: 10.3389/fmicb.2019.02061 (PMC6742758; doi:10.3389/fmicb.2019.02061)
Supplement: TABLE S4 — IMG/VR BLASTX homolog habitats. [file Data_Sheet_4.PDF]

Table S4: IMG/VR BLASTX Homologue Habitats

	Body Fluid Percentage	CSF Percentage
Curated Prophages	20.62	16.52
Oral	6.05	6.71
Digestive	8.75	7.78
Skin	0.18	0.83
Vagina	0.05	0.03
Pulmonary	0.56	0.39
Mammals	0.61	0.38
Birds	0.00	0.00
Invertebrates	0.00	0.01
Annelida	0.00	0.03
Cnidaria	0.00	0.03
Mollusca	0.28	0.24
Arthropoda	0.14	0.42
Aquatic	19.13	20.67
Fresh Water	0.05	0.15
Marine Water	0.00	0.05
Waste Water	3.40	3.55
Terrestrial	0.70	0.89
Soil	7.26	8.84
Plants	12.71	13.40
Air	0.00	0.03
Built Environment	7.82	9.51
Engineered	3.68	3.06
Bioreactor	0.56	0.41
Bioremediation	5.03	4.02
Solid Waste	0.93	1.08
Fungi	1.16	0.73
Bacteria	0.23	0.19
Microbial	0.05	0.05
Other	0.00	0.01
